# Limiting Resources Define the Global Pattern of Soil Microbial Carbon Use Efficiency

**DOI:** 10.1002/advs.202308176

**Published:** 2024-07-18

**Authors:** Yongxing Cui, Junxi Hu, Shushi Peng, Manuel Delgado‐Baquerizo, Daryl L. Moorhead, Robert L. Sinsabaugh, Xiaofeng Xu, Kevin M. Geyer, Linchuan Fang, Pete Smith, Josep Peñuelas, Yakov Kuzyakov, Ji Chen

**Affiliations:** ^1^ Institute of Biology Freie Universität Berlin 14195 Berlin Germany; ^2^ Department of Agroecology Aarhus University Tjele 8830 Denmark; ^3^ Sino‐French Institute for Earth System Science College of Urban and Environmental Sciences Peking University Beijing 100871 China; ^4^ College of Forestry Sichuan Agricultural University Chengdu 611130 China; ^5^ Laboratorio de Biodiversidad y Funcionamiento Ecosistémico. Instituto de Recursos Naturales y Agrobiología de Sevilla (IRNAS) CSIC, Av. Reina Mercedes 10 Sevilla E‐41012 Spain; ^6^ Department of Environmental Sciences University of Toledo Toledo OH 43606 USA; ^7^ Department of Biology University of New Mexico Albuquerque NM 87131 USA; ^8^ Biology Department San Diego State University San Diego CA 92182 USA; ^9^ Department of Biology Young Harris College Young Harris GA 30582 USA; ^10^ School of Resource and Environmental Engineering Wuhan University of Technology Wuhan 430070 China; ^11^ Institute of Biological and Environmental Sciences University of Aberdeen 23 St. Machar Drive Aberdeen AB24 3UU UK; ^12^ CSIC, Global Ecology Unit CREAF‐CSIC‐UAB Bellaterra Barcelona Catalonia 08913 Spain; ^13^ CREAF, 08913 Cerdanyola del Vallès Barcelona Catalonia 08193 Spain; ^14^ Department of Soil Science of Temperate Ecosystems Department of Agricultural Soil Science University of Goettingen 37077 Göttingen Germany; ^15^ Peoples Friendship University of Russia (RUDN University) Moscow 117198 Russia; ^16^ State Key Laboratory of Loess and Quaternary Geology Institute of Earth Environment Chinese Academy of Sciences Xi'an 710061 China; ^17^ Institute of Global Environmental Change Department of Earth and Environmental Science School of Human Settlements and Civil Engineering Xi'an Jiaotong University Xi'an Shaanxi Province 710049 China

**Keywords:** extracellular enzymatic activity, global change factors, microbial metabolisms, resource limitations, soil carbon cycling

## Abstract

Microbial carbon (C) use efficiency (CUE) delineates the proportion of organic C used by microorganisms for anabolism and ultimately influences the amount of C sequestered in soils. However, the key factors controlling CUE remain enigmatic, leading to considerable uncertainty in understanding soil C retention and predicting its responses to global change factors. Here, we investigate the global patterns of CUE estimate by stoichiometric modeling in surface soils of natural ecosystems, and examine its associations with temperature, precipitation, plant‐derived C and soil nutrient availability. We found that CUE is determined by the most limiting resource among these four basic environmental resources within specific climate zones (i.e., tropical, temperate, arid, and cold zones). Higher CUE is common in arid and cold zones and corresponds to limitations in temperature, water, and plant‐derived C input, while lower CUE is observed in tropical and temperate zones with widespread limitation of nutrients (e.g., nitrogen or phosphorus) in soil. The contrasting resource limitations among climate zones led to an apparent increase in CUE with increasing latitude. The resource‐specific dependence of CUE implies that soils in high latitudes with arid and cold environments may retain less organic C in the future, as warming and increased precipitation can reduce CUE. In contrast, oligotrophic soils in low latitudes may increase organic C retention, as CUE could be increased with concurrent anthropogenic nutrient inputs. The findings underscore the importance of resource limitations for CUE and suggest asymmetric responses of organic C retention in soils across latitudes to global change factors.

## Graphical abstract

This study investigates the global patterns of CUE and their links to fundamental environmental resources across climate zones. The study clarifies and theorizes resource limitations as the key mechanisms regulating CUE. Results underscore the importance of resource limitations for CUE and suggest asymmetric responses of organic C retention in soils across latitudes to global change factors.

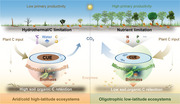

## 1 Introduction

Organic carbon (C) in soils, which originates from net primary production, is mainly decomposed by heterotrophic microorganisms that release CO_2_ back into the atmosphere.^[^ ^1^, ^2^ ^]^ The efficiency with which microorganisms assimilate organic C into biomass (commonly defined as C use efficiency, CUE) represents the balance between the accumulation and loss of organic C in soils and is critical for soil C storage and climate change mitigation.^[^ ^3^, ^4^, ^5^, ^6^ ^]^ Studies on this basic microbial characteristic, from cross‐system patterns to its responses to environmental change, have suggested that CUE could decline under future climate scenarios.^[^ ^7^, ^8^, ^9^ ^]^ However, the fundamental drivers of CUE in terrestrial ecosystems remain largely unknown,^[^ ^6^ ^]^ which hinders our understanding of the patterns and mechanisms governing CUE and, consequently, projections of soil C stocks under climate change.

The drivers of CUE are not clear for three main reasons. First, CUE is usually determined by incubation experiments in the specific context such as the methods (i.e., ^13^C‐/^14^C‐labeled substrates and ^18^O‐labeled water), experimental conditions (e.g., temperature and soil moisture), and duration (from hours to weeks).^[^ ^5^, ^10^, ^11^ ^]^ These differences complicate the discovery of robust spatial patterns of CUE and the corresponding drivers using either incubation experiments or meta‐analyses based on isotopic measurements.^[^ ^3^, ^4^ ^]^ Second, various environmental factors affecting CUE obscure the identification of determinants. CUE undoubtedly depends on numerous environmental conditions, given the constant and ubiquitous interactions of soil microorganisms with their environment.^[^ ^12^, ^13^ ^]^ Unfortunately, it is not possible to measure or assess the impacts of all possible factors on CUE. A crude alternative, generally used in current models of soil C cycling, is to represent the CUE by temperature‐dependent functions.^[^ ^9^, ^14^, ^15^ ^]^ This simplified method has identified broad spatial patterns of CUE,^[^ ^14^ ^]^ but only represents temperature effects and ignores other potential drivers. Third, varying impacts of drivers on CUE across environmental gradients may further obscure the mechanisms determining CUE. For example, temperature, soil water and nutrient availability, and nutrient stoichiometry have strong effects on CUE, but these effects depend on climate and ecosystem types.^[^ ^7^, ^16^, ^17^, ^18^ ^]^ The relative importance of the potential drivers of CUE under different environmental conditions is inconclusive.

Indeed, the resources required for microbial metabolism and the environmental factors that determine the availability of resources are potentially the most important drivers of CUE. Temperature, water, C source, and nutrients (mainly nitrogen (N) and phosphorus (P)) are the most important factors regulating microbial activity and growth.^[^ ^8^, ^12^, ^19^, ^20^, ^21^, ^22^ ^]^ These factors are also sensitive to global climate change. For example, climate change is affecting soil temperature and water availability, while atmospheric N deposition is disproportionately altering the relative availability of N and P in the soil across terrestrial ecosystems.^[^ ^23^, ^24^ ^]^ Identifying the effects of these fundamental factors on CUE under different environmental conditions is therefore a clear priority to understand the mechanisms controlling CUE and to predict the impact of global change on the microbially mediated soil C cycle.

**Figure 1:**
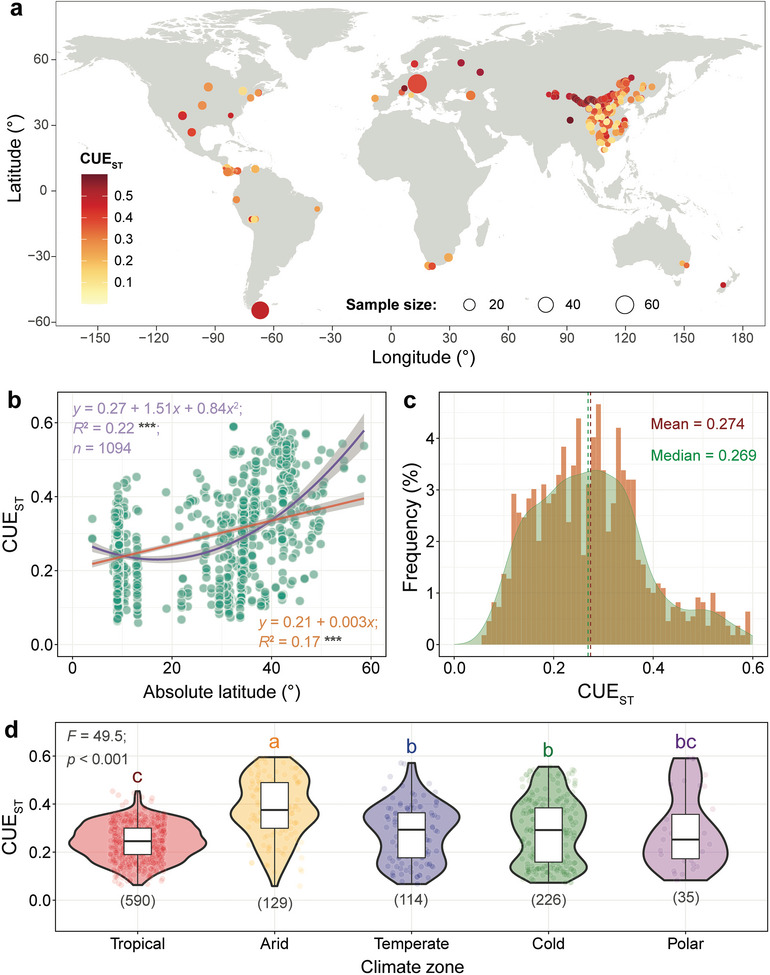
Global distribution of microbial carbon use efficiency (CUE_ST_) estimated by stoichiometric modeling. a), 1094 CUE_ST_ values at 447 sites from 160 studies. b), Latitudinal patterns of CUE_ST_ fitted by quadratic and linear models, ^***^ indicates *p* < 0.001 in the fitted model. c), Histogram of the frequency distribution of CUE_ST_ for the 1094 observations. d), Differences in CUE_ST_ among five climate zones. Tropical zone, CUE_ST_ mean = 0.243, standard deviation (SD) = 0.074; arid zone, mean = 0.381, SD = 0.126; temperate zone, mean = 0.281, SD = 0.117; cold zone, mean = 0.290, SD = 0.132; and polar zone, mean = 0.286, SD = 0.152. The numbers in parentheses below the violin plots indicate the sample size. Different letters indicate significant differences (*p* < 0.001) among the climate zones based on the analysis of linear mixed‐effect models followed by Tukey's tests.

To investigate patterns of CUE on a global scale, we estimated community‐level CUE (*n* = 1094) in natural ecosystems worldwide (**Figure** **1**a) using culture‐independent stoichiometric modeling.^[^ ^25^ ^]^ Compared to isotopic approaches based on incubation experiments, stoichiometric modeling can estimate CUE using measurable indicators relevant to microbial demand and supply of soil resources (details in Experimental Section). In particular, the CUE estimated by this method (hereafter “CUE_ST_”) embodies the ability of a microbial community in situ to reconcile disparities between resource availability and metabolic demand by obtaining C, N, and P from polymeric resources.^[^ ^5^, ^26^ ^]^ As such, it reflects physiological acclimatization, shifts in community composition, and genetic adaptations that regulate the production of ecoenzymes catalyzing polymeric resources. To explore which factors control CUE_ST_, we separately tested how CUE_ST_ is related to temperature, precipitation, plant‐derived C, and soil nutrients in tropical, arid, temperate, and cold zones (Figure S1, Supporting Information). The associations of CUE_ST_ with the four environmental factors were further verified using 43 isotope‐based manipulative experiments worldwide. We hypothesized that sufficient availability of C and water, along with a warm environment, would lead to low CUE_ST_, as microbial activity and overflow respiration are generally high under these conditions.^[^ ^27^ ^]^ In contrast, soil systems with a cold environment or low availability of C and water, combined with sufficient nutrients (e.g., N and P) would result in a high CUE_ST_.

## 2 Results

### 2.1 Global Patterns of CUE_ST_

CUE_ST_ increased with latitude across global natural ecosystems (*p* < 0.001, Figure 1b), despite a large variability (0.06‐0.59 with a mean of 0.27) (Figure 1a). CUE_ST_ also differed among five climate zones: tropical, arid, temperate, cold, and polar zones (*p* < 0.001, Figure 1d), with the lowest values in the tropical zone (mean of 0.24), and the highest in the arid zone (mean of 0.38).

### 2.2 Effects of Environmental Factors on CUE_ST_

Four environmental factors (mean annual precipitation, MAP; mean annual soil temperature, soil MAT; leaf area index, LAI; and soil pH) were considered as potential drivers of CUE_ST_. The four factors were supposed to represent water supply, temperature limitation, and the availability of plant‐derived C and soil nutrients (mainly N and P) for soil microorganisms, respectively (details in Experimental Section). Notably, soil pH rather than nutrient indicators was used to represent availability of soil nutrients to avoid statistical collinearity, as the stoichiometric model of CUE_ST_ includes soil N and P indicators as parameters (details in Experimental Section). We also combined the polar zone with the cold zone because the polar zone had a small sample size (35 observations) and the CUE_ST_ in the polar zone did not differ from the cold zone (*p* > 0.05, Figure 1d). Therefore, there were only four climate zones (i.e., tropical, arid, temperate, and cold zones) in the following analyses. The four environmental factors showed the expected gradients across climate zones (*p* < 0.001, Figure S3, Supporting Information). For example, MAP was lowest in the arid and cold zones, and both MAP and soil MAT were highest in the tropical zone.

**Figure 2:**
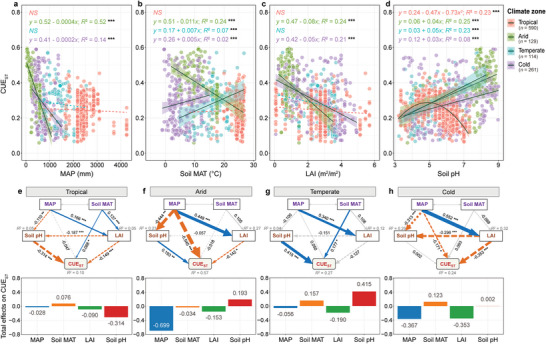
Effects of four environmental variables on microbial carbon use efficiency (CUE_ST_) in soils across climate zones. a–d) Generalized linear models identifying the relationships between the four environmental variables and CUE_ST_ for each climate zone. A quadratic model was used to identify the relationship between CUE_ST_ and soil pH for the tropical zone because the data showed an obvious unimodal pattern (d). e–h) Partial least squares path modeling of the major pathways of the influences of the four environmental variables on CUE_ST_ across the climate zones. A priori path modeling is shown in Figure S5 (Supporting Information). The upper part of each subplot shows the pathways of the influences of these environmental variables on CUE_ST_, and the lower part of each subplot shows the total effects of these variables on CUE_ST_. Blue solid and orange dotted arrows indicate positive and negative causal flows (*p* < 0.05), respectively. The numbers near the arrows indicate significant standardized path coefficients. *R* ^2^ indicates the variance of the dependent variable explained by the model. ^*^ *p* < 0.05; ^**^ *p* < 0.0; ^***^ *p* < 0.001. Gray solid/dashed arrows indicate the lack of significant relationships (effects) between the variables (*p* > 0.05). Note that the gray double‐sided arrows between MAP and soil MAT theoretically indicate potential interactions between them, as stated in our priori pathway modeling. However, we did not calculate the interactive values between them using our data in the PLS‐PM analysis, as the path in the PLS‐PM must be one‐way, representing the causal effect of one latent variable on another. MAP, mean annual precipitation; Soil MAT, mean annual soil temperature; LAI, leaf area index.

In the tropical and temperate zones with high primary productivity, precipitation, and temperature, soil pH had the strongest effect on CUE_ST_ compared to LAI, MAP, and soil MAT (*p* < 0.001, **Figure** **2**; Figure S3, Supporting Information). It is noteworthy that soil pH in the tropical zone correlated non‐linearly with CUE_ST_ (*p* < 0.001, Figure 2d), with CUE_ST_ being highest at a pH of 5.1 (*p* < 0.001, Figure S4, Supporting Information). However, LAI, MAP, and soil MAT were more important for CUE_ST_ in arid and cold zones with low primary productivity, precipitation, and temperature (*p* < 0.001, Figure 2a–c; Figure S3, Supporting Information).

The direct and indirect associations between these factors and CUE_ST_ were further assessed using partial least‐squares path modeling (Figure 2e–h; Tables S4‐S7; Figures S5, Supporting Information). We found that the direct effect of soil pH on CUE_ST_ was greater in both tropical (−0.31) and temperate (0.42) zones than in other zones, while the direct effects of MAP (−0.55) and LAI (−0.35) on CUE_ST_ were greater in arid and cold zones than in other zones. In addition, MAP had the greater indirect effects on CUE_ST_ compared to other factors in the arid (−0.15), temperate (−0.11), and cold (−0.20) zones (Tables S5‐S7, Supporting Information). Overall, soil pH had the largest total effects on CUE_ST_ in the tropical (−0.31) and temperate (0.42) zones (Figure 2e,g), while MAP had the largest total effects on CUE_ST_ in the arid (−0.70) and cold (−0.37) zones (Figure 2h). These results were further confirmed by the analysis of the selection of mixed‐effect models (Figure S6, Supporting Information) and random‐forest models (Figure S7, Supporting Information).

### 2.3 Response of Isotope‐Based CUE (CUE‐Isotope) to Changes in Environmental Factors

**Figure 3:**
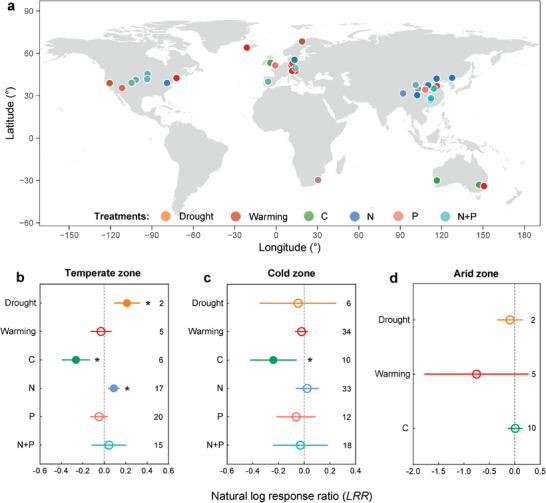
Responses of microbial carbon use efficiency (CUE‐isotope), measured with ^13^C‐ or ^18^O‐labeled approaches, to six manipulative factors worldwide. a), Site distribution of manipulative experiments used in this meta‐analysis (a total of 195 paired observations at 43 sites from 41 publications). Note that the yellow circles for the drought treatment are covered by the red circles in Europe, the United States, and China. b–d), Natural log response ratio (*LRR*) and 95% confidence intervals (*CIs*) of the CUE‐isotope on these manipulative factors for the temperate, cold, and arid zones. No experiment with N, P, and N+P additions met our criteria in the arid zone, and no experiment met our criteria for any manipulative factor in the tropical zone. Manipulative factors: drought (rainfall reduction), warming (soil warming), C (addition of plant litter), N (addition of mineral N), P (addition of mineral P), and N+P (addition of both mineral N and P). The numbers on the right side of the *CIs* indicate the sample sizes for each group. The effects of the manipulative factors were considered significant if the confidence interval did not include zero, that is, the filled circles with the asterisks (*) and the hollow circles indicate significant and non‐significant effects, respectively.

To verify the results of the statistical analyses, 195 paired observations of CUE‐isotope were collected at 43 sites from 41 publications around the world that independently measured CUE with either ^13^C‐labeled substrates or ^18^O‐labeled water (**Figure** **3**a). Selected studies with incubation periods are generally within 24 h to minimize the effects of trial duration on measurements. We quantified the responses of the CUE‐isotope to six manipulative factors to identify the drivers of the CUE‐isotope (Figure 3b–d). The responses of the CUE‐isotope to these manipulative factors are consistent with the effects of selected environmental factors on CUE_ST_ in different climate zones. On average across all studies and the six manipulative factors, the CUE‐isotope responded strongly to drought and C‐ and N‐additions in the temperate zone (Figure 3b), and to C‐addition in the cold zone (Figure 3d). For example, C‐addition decreased the CUE‐isotope in the cold zone, which is consistent with the results of the generalized linear model and partial least‐squares path modeling for CUE_ST_ (Figures 2c,h, and 3c). However, the CUE‐isotope did not respond to most manipulative factors (Figure 3), suggesting either low sensitivity or wide variation in microbial responses to environmental changes under incubation conditions.^[^ ^28^, ^29^, ^30^ ^]^

## 3 Discussion

**Figure 4:**
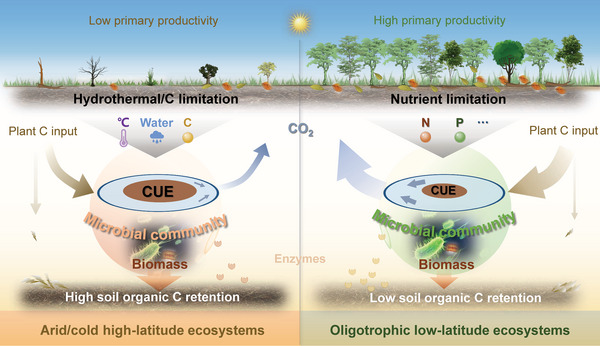
Conceptual framework showing the contrasting effects of different resource limitations on microbial carbon use efficiency (CUE) across climate zones. In this framework, the limitation of temperature, water, and plant C on microorganisms in the high latitudes leads to high CUE, which could thus contribute to the high potential of soil organic C retention in these ecosystems. In contrast, the limitation of soil nutrients such as nitrogen (N) or phosphorus (P) on microorganisms in the low latitudes results in low CUE, which could thus contribute to the low potential of soil organic C retention in these ecosystems, despite the much higher input of plant C.

The observed increase in CUE with latitude (Figure 1) supports our hypothesis that CUE would increase with increasing limitations in the availability of plant‐derived C and hydrothermal conditions, and vice versa. This latitudinal pattern of CUE was largely explained by selected MAP, soil MAT, LAI, and soil pH across four climate zones (Figures 2 and 3). We found that MAP has a greater effect on CUE in the arid and cold zones (generally high latitudes) than in other regions. These regions are generally characterized by limited hydrothermal conditions with low vegetation production. In general, the high availability of plant‐derived C with sufficient hydrothermal conditions in the soil can reduce CUE through increased microbial metabolic activity and overflow respiration.^[^ ^5^, ^21^ ^]^ A limited availability of plant‐derived C for soil microorganisms due to low vegetation production under limiting hydrothermal conditions in the arid and cold zones thus corresponded to high CUE (Figure 2c; Figure S3, Supporting Information). Limited soil water availability and low temperature could further contribute to the maintenance of high CUE (Figure 2a,b) by suppressing microbial metabolic rates.^[^ ^21^ ^]^ As a result, the combined limitations in plant‐derived C, soil water, and temperature contributed to high CUE in high‐latitude soils (**Figure** **4**).

In contrast, we found that soil pH had greater effects on CUE than MAP, soil MAT or LAI in both tropical and temperate zones (generally low latitudes). High availability of nutrients such as N and P in the soil tends to increase CUE by supporting the synthesis of microbial biomass.^[^ ^16^, ^30^ ^]^ However, low‐latitude soils are usually characterized by severe nutrient (especially P) deficiency due to the old age of the exposed parent material, heavy leaching of nutrients, and strong competition between microorganisms and plants for nutrients.^[^ ^31^, ^32^ ^]^ Such limitation in nutrient resources could therefore reduce CUE to maintain stoichiometric homeostasis of microbial biomass in low latitudes.^[^ ^26^ ^]^ High plant‐derived C input and favorable hydrothermal conditions in low latitudes could further reduce CUE by increasing microbial activity.^[^ ^1^ ^]^ Consequently, the limitation in soil nutrients could be the main reason for the low CUE in oligotrophic low latitudes.

Carbon and nutrients as crucial components of microbial metabolism and production directly regulate CUE. Low CUE in highly productive ecosystems and vice versa (Figure 2c) indicates that litter‐rich soils sequester organic C less efficiently than litter‐poor soils, especially in low latitudes with limited availability of nutrients. This is consistent with the litter decomposition model,^[^ ^33^ ^]^ which suggests that the transfer of C from plants to soil is less efficient in more productive ecosystems. Moreover, surface soils, which generally have a much higher input of plant litter with high C:N and C:P ratios, have a lower CUE (mean of 0.27, Figure 1a) compared to deeper soils (mean of CUE > 0.33).^[^ ^34^ ^]^ Such a pattern suggests microbial efforts to minimize the stoichiometric mismatch between supply and demand for maintaining homeostasis.^[^ ^35^, ^36^ ^]^ With an average global CUE of 0.27 for surface soils (Figure 1a), more than two‐thirds of plant‐derived C could return to the atmosphere via microbial metabolism in surface soils. A higher input of plant litter would consequently lead to a higher CO_2_ release from surface soils, which could also increase C loss from deeper soils through the priming effect.^[^ ^37^ ^]^ This counterbalance between microbial C retention and plant litter input limits the soil C sinks and may be an inherent constraint for sequestering more C in the soil.

In addition to the contrasting roles of C and nutrients in CUE, inconsistent effects of soil MAT on CUE were also observed in different climate zones (Figure 2b).^[^ ^9^, ^25^, ^38^ ^]^ The inconsistent effects likely result from two aspects. First, according to Liebig's Law of the Minimum, the most limiting factor in the environment probably has the greatest effect on microorganisms.^[^ ^21^ ^]^ For example, low water availability should have the dominant effect on microbial activity in the arid zone, while low temperature should have the dominant effect on microbial activity in the cold zone. Second, the differential impacts of temperature on vegetation production among climate zones (Figure 2e–h) could alter the relative importance of temperature for microbial activity and metabolism in different climate zones. Overall, the inconsistent effects of temperature on CUE across climate zones emphasize the uncertainties in predicting CUE in ecosystems based on functions that rely solely on individual variables.^[^ ^9^, ^39^ ^]^

## 4 Implications for Soil C Cycling and Uncertainties

The latitudinal patterns of CUE (Figure 1) indicate that the potential for soil organic C retention increases with latitude.^[^ ^6^, ^12^ ^]^ However, the resource dependence of CUE implies that the existing patterns of CUE in high and low latitudes could shift in opposite directions under global change. Increases in temperature, precipitation, and primary production could reduce CUE in high‐latitude soils by mitigating the limitations of temperature, water, and plant‐derived C on microorganisms and promoting their activity and overflow respiration.^[^ ^23^, ^40^ ^]^ In contrast, CUE could increase in low‐latitude soils if anthropogenic nutrient inputs such as N and P deposition increase the availability of soil nutrients,^[^ ^41^, ^42^ ^]^ which stimulate microbial anabolism and growth.^[^ ^43^, ^44^ ^]^ This argument is supported by a widely observed decrease in soil respiration and an increase in soil C sequestration under N deposition.^[^ ^45^, ^46^ ^]^ The opposite shifts in CUE across latitudes suggest that microbially mediated soil C retention may respond asymmetrically to global change factors, which is neither recognized nor accounted for in most biogeochemical models.

These results contribute to the understanding and prediction of the soil C cycle, but with several caveats. First, in our study, the CUE_ST_ estimated by the stoichiometric model and the CUE‐isotope measured by incubation experiments are from different ecosystems with various environmental conditions, which implies uncertainties when comparing their drivers. Additionally, we estimated a global average CUE_ST_ of 0.27 (Figure 1a), which was lower than the results measured with isotopic approaches (global mean = 0.37 under N addition, mean = 0.33 under control treatment).^[^ ^4^, ^6^ ^]^ This discrepancy could be partly because only a few enzymes were used as model parameters for estimating CUE_ST_. While this approach is rational (see Experimental Section). it may not fully capture microbial demand and actual resource acquisition.^[^ ^5^, ^47^ ^]^ We thus acknowledge the importance and necessity of comparing CUE from model estimates and isotope measurements as a priority in future research to reduce uncertainties in conclusions due to different methods.

Second, the enzymatic indicators we selected are generally microbial proxies using polymeric matter.^[^ ^26^, ^48^ ^]^ Soluble resources that do not require enzymatic catalysis for acquisition may skew predictions.^[^ ^5^, ^49^, ^50^ ^]^ However, we only used observations of surface soils (mean depth of 11.0 cm, Figure S2, Supporting Information) from natural ecosystems in which the dominant resource pools should be polymers from plant litter.^[^ ^51^ ^]^ This selection could reduce the uncertainties arising from soluble resources, such as those present in the rhizosphere and cropland.

Third, we considered only four environmental factors as potential drivers of CUE, whereas other abiotic factors (e.g., soil salinity and availability of iron and manganese) may also indirectly affect CUE by acting on microbial taxa and metabolic processes.^[^ ^10^, ^52^ ^]^ In particular, the use of soil pH to represent the availability of soil nutrients introduced uncertainties, as soil pH has varying effects on the availability of soil N and P, and others. For example, from acidic to alkaline, it determines the relative availability of NO_3_^−^ and NH_4_^+^,^[^ ^31^, ^53^ ^]^ while its effect on P availability is nonlinear.^[^ ^54^ ^]^ As found in our study, soil pH has a nonlinear effect on CUE in tropical soils, with the highest CUE observed at a pH of 5.1 (*p* < 0.001, Figure 2d; Figure S4, Supporting Information). This suggests either the crucial role of P availability in CUE in tropical soils due to widespread P limitation for microorganisms at low latitudes,^[^ ^43^ ^]^ or that microorganisms have different optimal pH ranges under varying environmental conditions. Since soils in tropical ecosystems are generally acidic, the optimal pH for microorganisms in these soils could be below neutral, such as the pH of 5.1 observed in our study. Moreover, soil pH can affect microbial community composition and metabolic processes by inducing physiological stress in microbial cells when pH deviates significantly from neutral.^[^ ^55^, ^56^ ^]^ These possible mechanisms suggest that soil pH plays varying roles in CUE across environmental gradients through both direct and indirect effects, which are not yet fully understood.

In summary, our findings are based on robust generalizations of CUE_ST_ over surface soils globally, verified by independent isotope‐based manipulative experiments. Beyond the debate on the environmental factors affecting CUE,^[^ ^4^, ^9^, ^47^, ^57^ ^]^ our study clarifies the fundamental drivers of CUE across climate zones and theorizes resource limitations as the key factors regulating CUE (Figure 4). Microbially mediated soil C turnover is one of the most intractable challenges in predicting the terrestrial C cycle.^[^ ^12^, ^52^, ^58^ ^]^ This understanding of the fundamental but distinct roles of plant‐derived C, hydrothermal conditions, and soil nutrients in CUE is a valuable step toward representing the complex soil C cycle with measurable indicators in biogeochemical models.

## 5 Experimental Section

### Data Collection—Global Data of the Model Parameters for the Estimation of CUE_ST_

We compiled a global dataset of ecoenzymatic activities (EEA), microbial biomass, and soil nutrients in surface soils (mean depth ≈ 11 cm, Figure S2, Supporting Information) from a literature search in the Web of Science (http://isiknowledge.com) and the Google Scholar Resource Integrated Database (https://scholar.google.com). Studies published between 1980 and 2022 were searched for using combinations of the keywords “extracellular enzymes OR exoenzymes OR ecoenzymes”, “threshold element ratio OR enzyme stoichiometry modeling”, and “microbial biomass OR microbial resource limitations”. The criteria for inclusion were as follows. First, the studies included: 1) the activities of C‐, N‐, and P‐acquiring enzymes, i.e., β−1, 4‐glucosidase (BG) as C‐acquiring enzyme, β−1, 4‐N‐acetylglucosaminidase (NAG) and/or L‐leucine aminopeptidase (LAP) as N‐acquiring enzymes, and acid or alkaline phosphatase (AP) as P‐acquiring enzymes (Table S1, Supporting Information), 2) the concentrations of microbial biomass C (MBC), N (MBN), and P (MBP), and 3) the concentrations of C (soil organic C, SOC), N (soil total N, TN) and P (soil total P, TP), as these indicators are the parameters of the stoichiometric model needed to estimate CUE_ST_.^[^ ^26^ ^]^ Second, EEA was measured fluorometrically with a 200‐µm solution of substrates labeled with 4‐methylumbelliferone or 7‐amino‐4‐methylcoumarin. Third, microbial biomass was determined by chloroform fumigation extraction. Fourth, data from intensively managed ecosystems (e.g., agroforestry, fertilized plantations, sown pastures, cropland, and urban forests) were excluded to avoid unexpected anthropogenic disturbances.

Based on these criteria, we selected 1094 paired observations of terrestrial surface soils at 477 geographic locations from 160 studies (Figures S1 and S2, Supporting Information). Data from the selected studies were extracted from tables or figures using GetData Graph Digitizer v.2.25. We also extracted the corresponding location information (longitude and latitude) from the literature. The locations of these sampling sites were initially divided into five climate zones (tropical, arid, temperate, cold, and polar zones) based on the global Köppen‐Geiger grid map of climate classification.^[^ ^59^ ^]^ However, we further combined the polar zone with the cold zone, because the polar zone has few observations (*n* = 35) and similar climate conditions to the cold zones, especially for MAT, the main limiting factor.

### Environmental Variables

We considered four environmental variables (MAP, soil MAT, LAI, and soil pH) as potential drivers of CUE_ST_, as they define the basic conditions for microbial survival and reproduction,^[^ ^12^, ^20^, ^21^ ^]^ Specifically, MAP, soil MAT, LAI, and soil pH were considered to represent water supply, temperature limitation, and availability of plant‐derived C and nutrients (mainly N and P) to soil microbial communities, respectively. Soil temperatures are generally less variable than atmospheric temperatures, which are derived from estimates of air temperature.^[^ ^60^ ^]^ We have therefore used the data for soil MAT from a recent study instead of the mean annual atmospheric temperature.^[^ ^60^ ^]^ We used the LAI as an index for the availability of plant‐derived C for two reasons. First, almost all C sources in surface soils originally come from the input of plant litter. Second, it was recently found that plant litter, rather than soil organic matter processed by microorganisms, is the dominant C source for microbial metabolism.^[^ ^19^ ^]^ In addition, the soil N and P variables were not used to directly represent nutrient availability in the soil, as the stoichiometric model used includes these variables as parameters. Soil pH is a fundamental regulator of N and P availability and indirectly represents their supply.^[^ ^61^, ^62^ ^]^ We therefore used soil pH as an indicator of soil N and P availability. We retrieved MAP, soil MAT, and LAI from different sources with a relatively fine spatial resolution (see Table S3, Supporting Information for details). Soil pH data were collected from the 160 studies we screened. Sixteen studies did not contain soil pH data (82 values). These missing values were extracted from other recently published studies with the same sample location information and similar geographic coordinates. In general, soil P availability is limited in the tropical zone, and N availability is typically low in the temperate zone. The arid zone is characterized by low precipitation and plant productivity, while cold zone exhibits low temperature and plant productivity. Consequently, nutrients in tropical and temperate zones, water and plant‐derived C in arid zone, and temperature and plant‐derived C in cold zone should be the primary limiting resources for soil microorganisms.

### Dataset for CUE‐Isotope‐Based on ^13^C or ^18^O Labeled Approaches

We compiled another global dataset on the responses of ^13^C/^18^O‐based CUE to various manipulative factors. Specifically, we considered six treatments: reduction of precipitation (simulation of drought), soil warming, addition of plant litter, addition of mineral N, addition of mineral P, and addition of both mineral N and P. These treatments corresponded to the four environmental factors we selected. Relevant studies were identified by searching the Web of Science and the Google Scholar Resource Integrated Database using combinations of the keywords “carbon use efficiency OR growth efficiency” and “soil microbial OR soil microbe” and “carbon addition OR nitrogen addition OR phosphorus addition OR fertilization OR warming OR elevated temperature OR drought OR decreased precipitation”. The studies had to fulfill the following criteria: 1) experimental site, vegetation, and soil type were similar in the controls and treatments, and 2) the mean values of the variables could be extracted. A total of 195 paired observations were obtained from 43 sites around the world in 41 publications that met these criteria (Figure 3a). For each study, we extracted the CUE‐isotope values in the controls and treatments from the tables or figures using Engauge Digitizer 4.1 (Free Software Foundation, Inc., Boston, USA). These 41 studies (see reference list in the Supporting Information) are not included in the 160 studies used to estimate the CUE_ST_.

### Estimating Community‐Level CUE_ST_—Comparison of Isotope‐based Approaches and Stoichiometric Modeling for the Characterization of CUE

Current approaches to quantify CUE in soil are based on different assumptions and represent different aspects of microbial C metabolism.^[^ ^5^, ^47^ ^]^ The use of ^13^C/^14^C labeled substrates quantifies the incorporation of C from substrates into the microbial biomass and the release of C by microbial respiration.^[^ ^4^, ^5^ ^]^ Similarly, substrate‐independent approaches measure the incorporation of ^18^O from water into DNA simultaneously with the loss of C through respiration to estimate CUE.^[^ ^4^, ^57^ ^]^ These isotope‐based approaches identify key processes of microbial anabolism and provide irreplaceable methods for direct measurement of CUE. However, any disturbance and addition of resources in incubation experiments could alter the structures and functions of the original communities and trigger cascading responses of microbial metabolism to other resources.^[^ ^63^, ^64^ ^]^ These uncertainties make it difficult to identify general patterns of CUE across ecosystems, whether through multi‐ecosystem research or meta‐analyses involving isotope‐based incubation experiments.

Alternatively, enzyme‐based stoichiometric modeling can estimate CUE independently of incubation experiments.^[^ ^5^, ^25^, ^26^, ^65^ ^]^ With this approach, measurable indicators of natural communities can be used to estimate CUE, making the estimated CUEs highly comparable among studies. Despite the obvious advantage, CUE estimates at a large scale based on field studies or meta‐analyses using this method are rare, because the parameters of the model contain many indicators that need to be measured.^[^ ^26^, ^34^, ^65^ ^]^ In this study, we collected 160 individual studies on a global scale that contain all the indicators needed to estimate CUE_ST_ with this model. Thus, we were able to use this stoichiometric method to estimate CUE_ST_ across terrestrial ecosystems.

### Stoichiometric Modeling for the Estimation of CUE_ST_

Sinsabaugh & Follstad Shah^[^ ^26^ ^]^ proposed the enzyme‐based stoichiometric model that incorporates EEA, microbial biomass, and soil resources to estimate CUE at the community level. The basis of this stoichiometric method is the use of several specific EEAs to represent the microbial resource requirements. In particular, soil microorganisms synthesize and excrete ecoenzymes (Table S1, Supporting Information) that degrade various organic macromolecules into products available for microbial assimilation. The EEA profile represents the relative microbial acquisition of C, N, and P from polymeric sources to meet microbial demand and maintain stoichiometric homeostasis of microbial biomass.^[^ ^26^ ^]^ Many ecoenzymes contribute to the degradation of complex polymers (e.g., cellulose), but only a few (i.e., BG, NAG and/or LAP, and AP) catalyze the terminal reactions of the most common substrates and produce soluble products for microbial assimilation. These ecoenzymes thus define the functional interface between product release and microbial acquisition.^[^ ^66^ ^]^ They generally have the highest activities per unit of microbial biomass and are strongly associated with litter decay and microbial metabolism compared to other ecoenzymes.^[^ ^48^ ^]^ As a result, these ecoenzymes are usually selected as the proximate agents of microbial nutrient acquisition during metabolism.^[^ ^26^, ^67^ ^]^

We estimated CUE_ST_ using the following Equations ((1), (2), (3)):^[^ ^25^ ^]^

(1) $$CUE_{ST}=CUE^{max}\;\times {\left\{\frac{\left(S_{C:N}\times S_{C:P}\right)}{\left[\left(K_{C:N}+S_{C:N}\right)\times \left(K_{C:P}+S_{C:P}\right)\right]}\right\}}^{0.5}$$

(2) $$S_{C:N}=\frac{B_{C:N}}{L_{C:N}}\;\times \frac{1}{EEA_{C:N}}$$

(3) $$S_{C:P}=\frac{B_{C:P}}{L_{C:P}}\;\times \frac{1}{EEA_{C:P}}$$

where *S*_*C*:*N*_ and *S*_*C*:*P*_ are scalars representing the extent to which the allocation of EEA offsets the disparity between the elemental composition of available resources and the composition of microbial biomass. In this case, *EEA*_*C*:*N*_ and *EEA*_*C*:*P*_ were calculated as BG/(NAG + LAP) and BG/AP, respectively. In addition, L_C:N_ and L_C:P_ were calculated as molar ratios of SOC:TN and SOC:TP, respectively, and B_C:N_ and B_C:P_ were calculated as molar ratios of MBC:MBN and MBC:MBP. Both *K*_*C*:*N*_ and *K*_*C*:*P*_ are half‐saturation constants for CUE_ST_ based on the availability of C, N, and P, and are assumed to be 0.5.^[^ ^26^ ^]^ *CUE^max^* (maximum CUE) is 0.6, based on metabolic kinetics and energetics.^[^ ^26^, ^68^, ^69^ ^]^ Finally, it was found that the estimated CUE_ST_ was normally distributed on a global scale (Anderson‐Darling normality test, *A* = 0.21 and *p* = 0.86; Figure 1c).

### Meta‐Analysis

We conducted a meta‐analysis to obtain data on the responses of CUE‐isotope to six manipulative factors on a global scale.^[^ ^70^ ^]^ The dataset for the analysis includes means, standard deviations (*SD_s_*), and sample sizes (*n*) extracted from published studies. When the standard error (*SE*) was reported instead of the *SD*, the *SD* was calculated as follows:

(4) $$SD=SE\sqrt{n}$$

When neither *SD* nor *SE* was reported, we approximated the missing *SD* by multiplying the reported mean by the average coefficient of variance of the full dataset. We approximated the *SD_s_* for the control and the treatments separately.

The responses of CUE‐isotope to these treatments were evaluated using the natural logarithm of the response ratio (*LRR*):

(5) $$LRR=ln\;\left(\frac{\bar{X_{t}}}{\bar{X_{c}}}\right)=ln\left(\bar{X_{t}}\right)-ln\left(\bar{X_{c}}\right)$$

where $\bar{X_{t}}$ and $\bar{X_{c}}$ are the means for the treatments and controls, respectively. The variance (*ν*) of the *LRR* was calculated as:

(6) $$\nu =\frac{S_{t}^{2}}{n_{t}{\bar{X_{t}}}^{2}}+\frac{S_{c}^{2}}{n_{c}{\bar{X_{c}}}^{2}}$$

where *n_t_* and *n_c_* are the sample sizes for the treatments and controls, respectively, and *S_t_* and *S_c_* are the standard deviations for the treatments and controls, respectively.

The 95% confidence interval (CI) for *LRR* was calculated as:

(7) 95%CI=LRR±1.96s×LRR

We used the “rma.mv” function in the R package “metafor”^[^ ^71^ ^]^ to calculate the weighted mean response ratio and 95% confidence intervals (CIs) to quantify the response of the CUE‐isotope to these selected treatments. The study site was used as a random factor when measuring the weighted mean response ratio and 95% CIs. The effect was considered significant if the 95% CIs did not overlap with zero. In addition, publication bias was tested with a funnel plot (Figure S8, Supporting Information), and the observed pattern indicated no evidence of publication bias across climate zones.

### Statistical Analysis

We used both quadratic and linear models to describe the latitudinal pattern of CUE_ST_ (Figure 1b). After a normality test for the estimated CUE_ST_ (*n* = 1094) using the Anderson‐Darling normality test, the linear mixed‐effects models followed by Tukey's tests were used to identify the differences in CUE_ST_ (Figure 1d) and four environmental variables among climate zones (Figure S3, Supporting Information). This model was constructed using the “lme” function in the “nlme” package, with “climate zone” and “vegetation type” as fixed factors and “sampling site” as a random factor.

Generalized linear models examined the relationships between CUE_ST_ and the four environmental variables (MAP, soil MAT, LAI, and soil pH) in the different climate zones (Figure 2a–d). The quadratic model was used to identify the relationship between CUE_ST_ and soil pH in the tropical zone, as there was an obvious nonlinear trend between the two variables (Figure 2d). We also identified the pH threshold using a piecewise linear‐regression analysis (Figure S4; Table S2, Supporting Information). The relationship between CUE_ST_ and soil pH was fitted and tested with linear models using the “segmented” package.^[^ ^72^ ^]^ Confidence intervals of thresholds (breakpoints) were calculated based on 1000 bootstrap samples using the “SiZer” package.^[^ ^73^ ^]^ We investigated the cascading relationships between the four environmental variables and CUE_ST_ to identify the direct and indirect effects of water, temperature, plant‐derived C, and soil nutrients on CUE_ST_. Based on the priori path model (Figure S5, Supporting Information), we applied partial least squares path modeling to identify significant pathways where the variables had a substantial effect on CUE_ST_. After standardizing the raw data using the “standardize” function, we constructed the models using the “innerplot” function in the “plspm” package with default parameters.^[^ ^74^ ^]^ The detailed results of the final modeling for each climate zone, including the direct and indirect effects of the variables on the CUE_ST_, were presented in Tables S4‐S7 (Supporting Information).

We also took two additional approaches to determine the importance of the four environmental variables in affecting CUE_ST_ for each climate zone. First, an analysis of mixed‐effects model selection was performed to identify the most important predictors among the four environmental variables affecting CUE_ST_ using the “glmulti” package.^[^ ^75^ ^]^ Model selection was based on maximum‐likelihood estimation. The importance of each predictor was calculated as the sum of the Akaike weights for the models containing that predictors (Figure S6, Supporting Information). A cutoff value of 0.8 was set to distinguish between essential and unessential predictors.^[^ ^75^ ^]^ Second, we assessed the effects of the four environmental variables on CUE_ST_ by training random‐forest models for each climate zone using the “RandomForest” package (Figure S7, Supporting Information). A 10‐fold cross‐validation was performed to determine the best models and the potential importance of the variables using the “rfcv” function. The significance of the model and the cross‐validated *R* ^2^ were assessed using 1500 permutations of the response variables with the “A3” package. We calculated the increase in mean squared error (MSE) (%) for each potential predictor in the constructed random‐forest models to determine the relative importance of all environmental variables in explaining the variations of CUE_ST_ using the “rfPermut” package. Similarly, the significance of each predictor for the response variable was assessed using the “rfPermute” package. All statistical analysis were performed using R software (v.3.3.2).^[^ ^76^ ^]^

## Conflict of Interest

The authors declare no conflict of interest.

## Author Contributions

J.H. should be considered joint first authors. Y.C. conceived and developed the study. Y.C. and J.H. compiled the data sets. Y.C. and J.H. performed data analysis. Y.C., J.H., S.P., M.D.B., D.L.M., R.L.S., and Y.K. contributed to the interpretation of the results. Y.C., J.H., S.P., M.D.B., D.L.M., and R.L.S., X.X., P.S., J.P., Y.K., and J.C. discussed the results and wrote and revised the manuscript.

## Supporting information

Supporting Information

Supporting Information

Supporting Information

## Data Availability

The data that supports the findings of this study are available in the supplementary information of this article.
