# Supplementary material for: Limiting Resources Define the Global Pattern of Soil Microbial Carbon Use Efficiency
Source: Adv Sci (Weinh). 2024 Jul 18;11(35):2308176. doi: 10.1002/advs.202308176 (PMC11425281; doi:10.1002/advs.202308176)
Supplement: Supplementary file 1 — Supporting Information [file ADVS-11-2308176-s003.pdf]

Limiting Resources Define the Global Pattern of Soil Microbial Carbon Use Efficiency

Yongxing Cui [1, 2, 3, 18 *], Junxi Hu [4, 18], Shushi Peng [3], Manuel Delgado-Baquerizo [5], Daryl L. Moorhead [6], Robert L. Sinsabaugh [7], Xiaofeng Xu [8], Kevin Geyer [9], Linchuan Fang [10], Pete Smith [11], Josep Peñuelas [12, 13], Yakov Kuzyakov [14, 15], Ji Chen [2, 16, 17 *]

[1] Institute of Biology, Freie Universität Berlin, Berlin 14195, Germany
[2] Department of Agroecology, Aarhus University, Tjele 8830, Denmark
[3] Sino-French Institute for Earth System Science, College of Urban and Environmental Sciences, Peking University, Beijing, 100871, China
[4] College of Forestry, Sichuan Agricultural University, Chengdu, 611130, China
[5] Laboratorio de Biodiversidad y Funcionamiento Ecosistémico. Instituto de Recursos Naturales y Agrobiología de Sevilla (IRNAS), CSIC, Av. Reina Mercedes 10, E-41012, Sevilla, Spain
[6] Department of Environmental Sciences, University of Toledo, Toledo, OH, 43606, USA
[7] Department of Biology, University of New Mexico, Albuquerque, NM, 87131, USA
[8] Biology Department, San Diego State University, San Diego, CA, 92182, USA
[9] Department of Biology, Young Harris College, Young Harris, GA, 30582, USA
[10] School of Resource and Environmental Engineering, Wuhan University of Technology, Wuhan, 430070, China
[11] Institute of Biological and Environmental Sciences, University of Aberdeen, 23 St. Machar Drive, Aberdeen AB24 3UU, UK
[12] CSIC, Global Ecology Unit CREAF-CSIC-UAB, Bellaterra, Barcelona, Catalonia, 08913, Spain
[13] CREAF, 08913 Cerdanyola del Vallès, Barcelona, Catalonia, 08193, Spain
[14] Department of Soil Science of Temperate Ecosystems, Department of Agricultural Soil Science, University of Goettingen, 37077, Göttingen, Germany
[15] Peoples Friendship University of Russia (RUDN University), Moscow, 117198, Russia
[16] State Key Laboratory of Loess and Quaternary Geology, Institute of Earth Environment, Chinese Academy of Sciences, Xi’an, 710061, China
[17] Institute of Global Environmental Change, Department of Earth and Environmental Science, School of Human Settlements and Civil Engineering, Xi’an Jiaotong University, Xi'an, Shaanxi Province, 710049, China
[18] Yongxing Cui and Junxi Hu should be considered joint first authors

[*] Corresponding authors:
Yongxing Cui (Email: cuiyongxing@zedat.fu-berlin.de)
Ji Chen (Email: Ji.chen@agro.au.dk)

Table and Figure captions

Table S1 Extracellular enzymes commonly used to represent microbial demand and acquisition of C, N, and P in the environments.

Table S2 Execution and results of the piecewise regression analyses for Fig. S4.

Table S3 Description of environmental variables used as potential drivers of microbial carbon use efficiency (CUE_{ST}).

Table S4 Detailed results of partial least squares path modeling for the tropical zone.

Table S5 Detailed results of partial least squares path modeling for the arid zone.

Table S6 Detailed results of partial least squares path modeling for the temperate zone.

Table S7 Detailed results of partial least squares path modeling for the cold zone.

Fig. S1 Global distribution of sample sites for estimating microbial carbon use efficiency (CUE_{ST}) using stoichiometry modeling.

Fig. S2 Kernel density plots showing the mean of soil depth in different climate zones.

Fig. S3 Boxplots showing the differences of four environmental variables among four climate zones for 1,094 observations on the global scale.

Fig. S4 Effects of soil pH on microbial carbon use efficiency (CUE_{ST}) in the tropical zone.

Fig. S5 The priori pathway modeling including the direct and indirect effects of the four environmental factors we selected on microbial carbon use efficiency (CUE).

Fig. S6 Effects of four selected environmental variables on microbial carbon use efficiency (CUE_{ST}) using the analysis of mixed-effects model selection across climate zones.

Fig. S7 Effects of four selected environmental variables on microbial carbon use efficiency (CUE_{ST}) using random-forest models across climate zones.

Fig. S8 Funnel plot testing the publication bias of the natural logarithmic response ratio of microbial carbon use efficiency (CUE-isotope) measured with ^{13}C- or ^{18}O-labeled approaches to six manipulative factors around the world.

Table S1

Extracellular enzymes commonly used to represent microbial demand and acquisition of C, N, and P in the environments.

Ecoenzyme	Abbreviation	EC[a]	Function
β-1, 4-glucosidase	BG	3.2.1.21	Cellulose degradation: hydrolyzes glucose from cellobiose
β-1, 4-N-acetylglucosaminidase	NAG	3.2.1.14	Chitin and peptidoglycan degradation: hydrolyzes glucosamine from chitobiose
L-leucine aminopeptidase	LAP	3.4.11.1	Proteolysis: hydrolyzes leucine and other hydrophobic amino acids from the N terminus of polypeptides
Acid or alkaline phosphatase	AP	3.1.3.1	Hydrolyzes phosphate from phosphosaccharides and phospholipids

Note: Enzyme commission classification (Sinsabaugh et al., 2008, 2009).

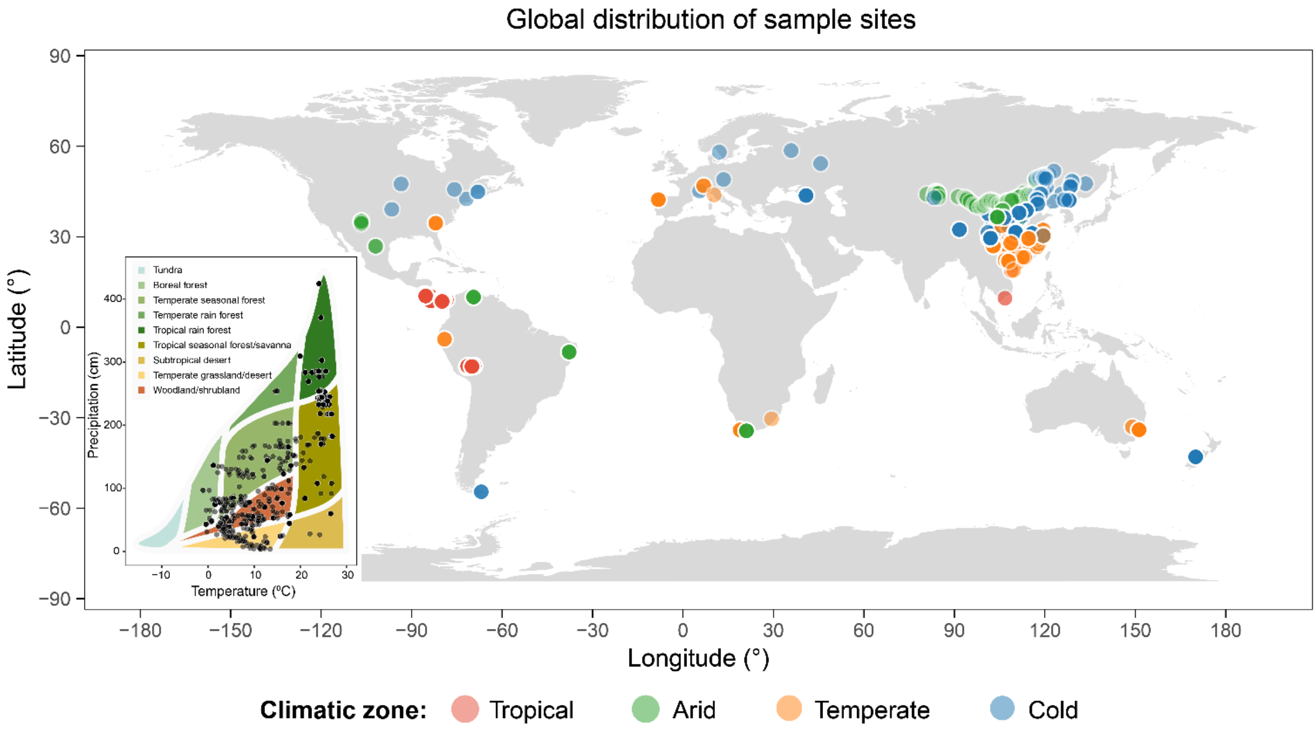

Fig. S1 Global distribution of sample sites for estimating microbial carbon use efficiency (CUE_{ST}) using stoichiometry modeling. A total of 1094 observations were collected at 447 sites from 160 studies.

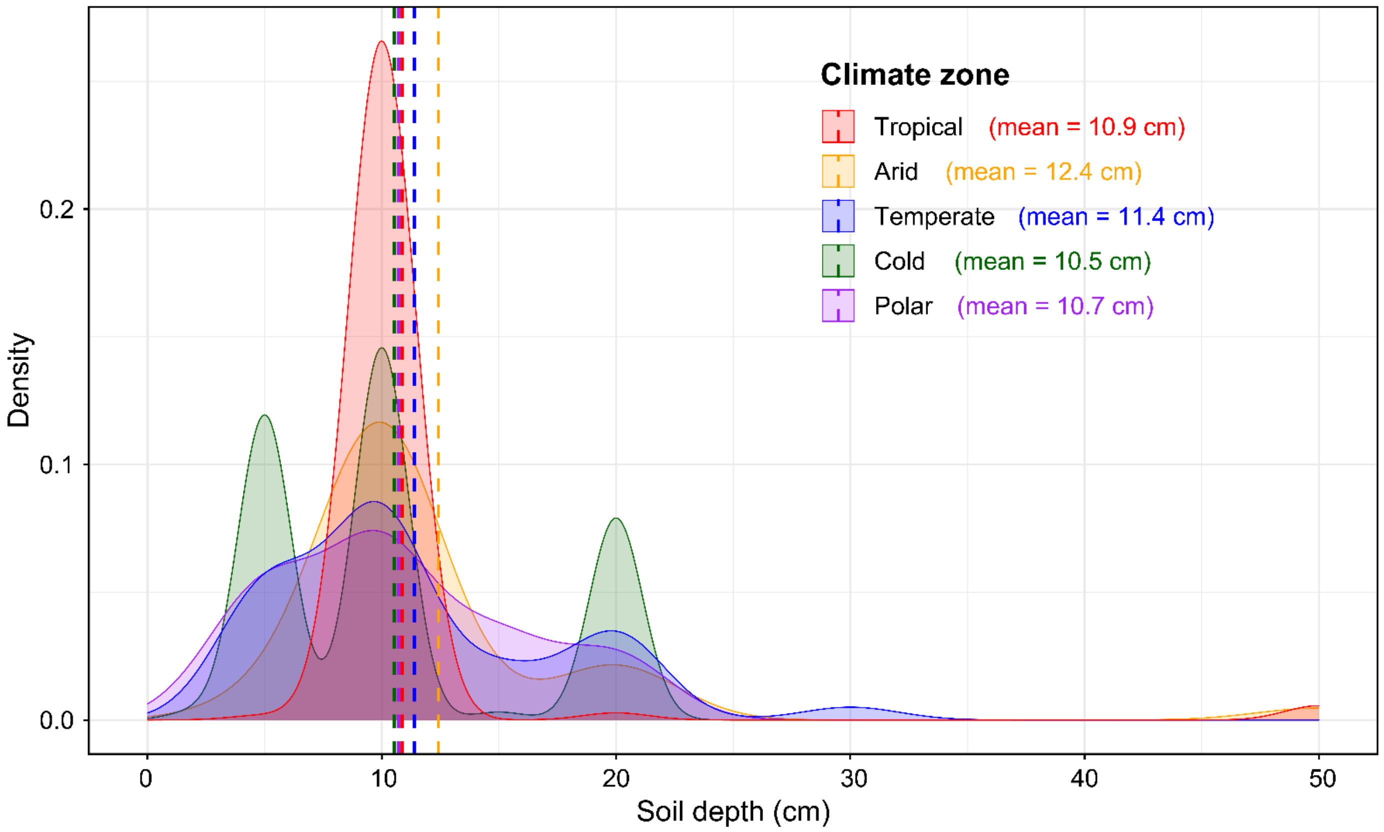

Fig. S2 Kernel density plots showing the mean of soil depth in different climate zones. The dashed lines indicate the mean of soil depth in each climate zone. The global mean of soil depth is 11.0 cm.

Table S2:

Execution and results of the piecewise regression analyses for Fig. S4.

First step:

Calculating the adjusted R^2 and P values of the piecewise linear regression using the "segmented" package

#Fitting a simple linear regression model between CUE_{ST} and Soil_pH first
fit_lm <- lm(CUE_{ST}~Soil_pH, data = df1)
summary(fit_lm)

#In the above-known linear regression model, the possible breakpoints were found by the "segmented" function.
> lm_seg1 <- segmented(fit_lm, seg.Z = ~ Soil_pH, npsi = 1)
> summary(lm_seg1)
Call:
segmented.lm(obj = fit_lm, seg.Z = ~ Soil_pH, npsi = 1)

Estimated Break-Point(s):

	Est.	St.Err
psi1. Soil_pH:	**5.116**	0.084

Meaningful coefficients of the linear terms:

	Estimate	Std. Error	*t*	Pr(>|t|)	Significance
(Intercept)	0.004678	0.041953	0.112	0.911	
Soil_pH	0.059697	0.009618	6.207	1.02e-09	***
U1. Soil_pH	-0.137583	0.011731	-11.728	NA	

***, $P < 0.001$; **, $P < 0.01$; *, $P < 0.05$
Residual standard error: 0.06432 on 586 degrees of freedom
Multiple R^2 = 0.2519, adjusted R^2 = **0.248**

Boot restarting based on 6 samples. Last fit:
Convergence attained in 2 iterations (rel. change 6.5171e-13)

Second step:

Calculating the confidence interval of piecewise linear regression using the "SiZer" package

#The breakpoint of the piecewise linear regression is automatically determined by the " piecewise.linear" function, and the 97.5% confidence interval is estimated using 1000 bootstraps.
> model <- piecewise.linear(x = df1$Soil_pH, y = df1$$CUE_{ST}$,
CI = TRUE, bootstrap.samples = 1000, sig.level = 0.05)
> model
Threshold alpha = **5.11556055804921**

CI	Change Point	Initial Slope	Slope Change	Second Slope
2.50%	**5.013063**	0.03128239	-0.1618992	-0.09408365
97.50%	**5.492486**	0.08005548	-0.1135175	-0.06638842

Table S3

Description of environmental variables used as potential drivers of microbial carbon use efficiency (CUE_{ST}).

Abbreviation	Variable	Unit	Source	Period/layer	Reference
MAP	Mean annual precipitation	mm	WorldClim2	1970-2000	Fick and Hijmans, 2017
Soil MAT	Mean annual soil temperature	℃	https://zenodo.org/record/4558732#.Y2N9XsiUdvJ	1979-2013/ 0-5 cm	Lembrechts et al., 2022
LAI	Leaf area index	m^2 m^{-2}	https://daac.ornl.gov/cgi-bin/dsviewer.pl?ds_id=1653	1981-2015	Mao and Yan, 2019
Soil pH	Soil pH	-	Collected this study	1980-2022	See the attached database

References:

Fick, S.E., & Hijmans, R.J. (2017). WorldClim 2: new 1-km spatial resolution climate surfaces for global land areas. *International Journal of Climatology*, 37(12), 4302-4315.

Lembrechts, J.J., van den Hoogen, J., Aalto, J., Ashcroft, M.B., De Frenne, P., Kemppinen, J., ... & Hik, D.S. (2022). Global maps of soil temperature. *Global Change Biology*, 28(9), 3110-3144.

Mao, J., & Yan, B. (2019). Global Monthly Mean Leaf Area Index Climatology, 1981-2015. *ORNL DAAC*.

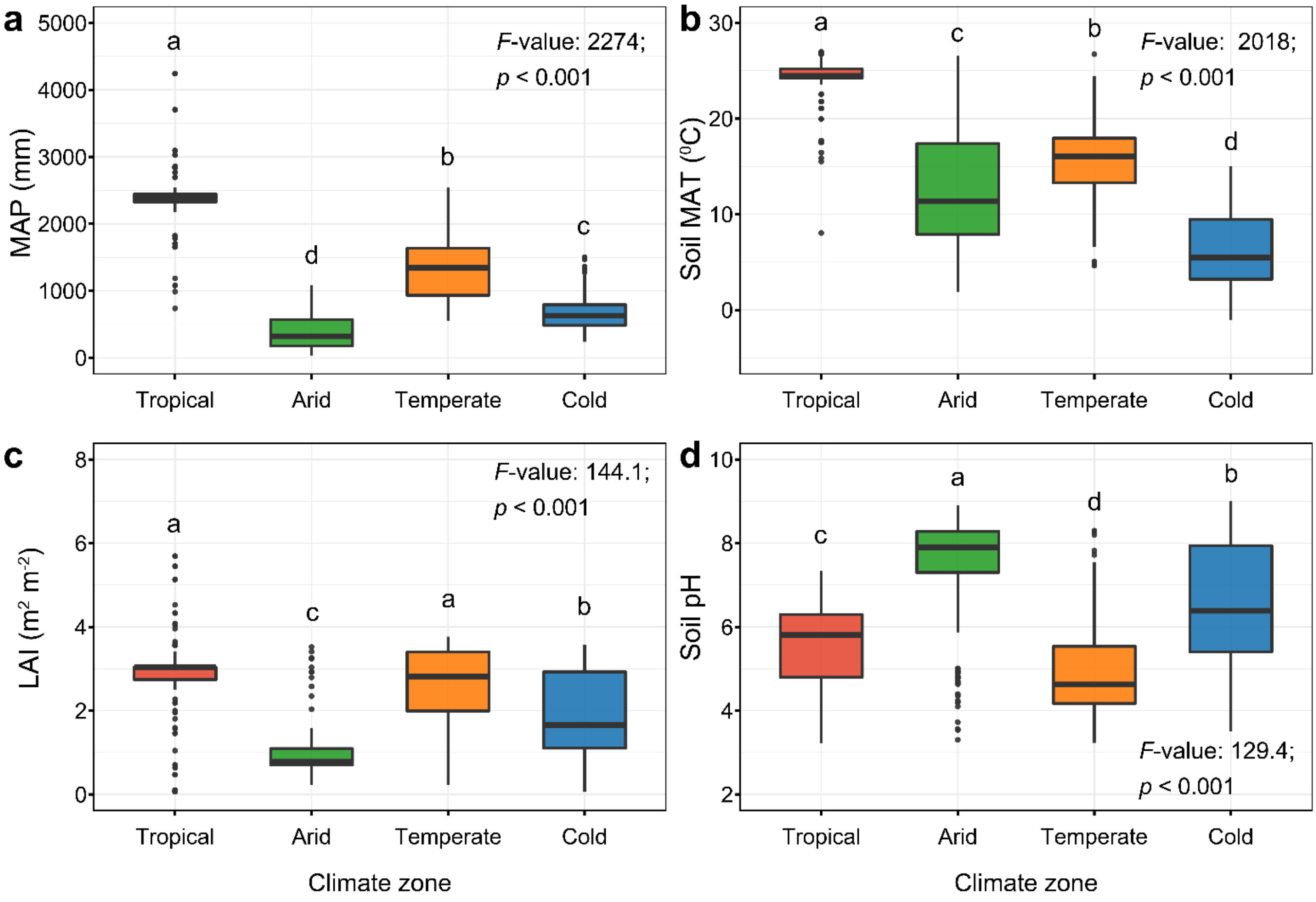

Fig. S3 Boxplots showing the differences of four environmental variables among four climate zones for 1,094 observations on the global scale. Letters indicate significant differences ($p < 0.001$) among climate zones based on a linear mixed-effects model analysis followed by Tukey's test. MAP, mean annual precipitation; Soil MAT, mean annual soil temperature; LAI, leaf area index.

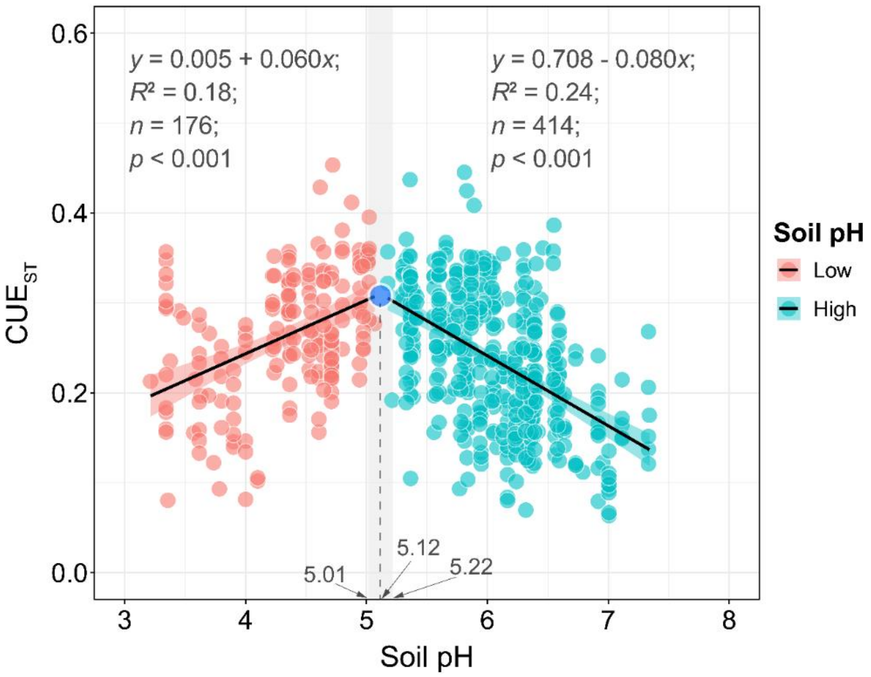

Fig. S4 Effects of soil pH on microbial carbon use efficiency (CUE_{ST}) in the tropical zone. The pH threshold was estimated using piecewise regression analyses, and correlations of CUE_{ST} with soil pH above and below the threshold were identified using generalized linear models. The shaded circles indicate the threshold, and the shaded areas are the 97.5% confidence intervals of the threshold. The solid black lines show the model fits between CUE_{ST} and soil pH.

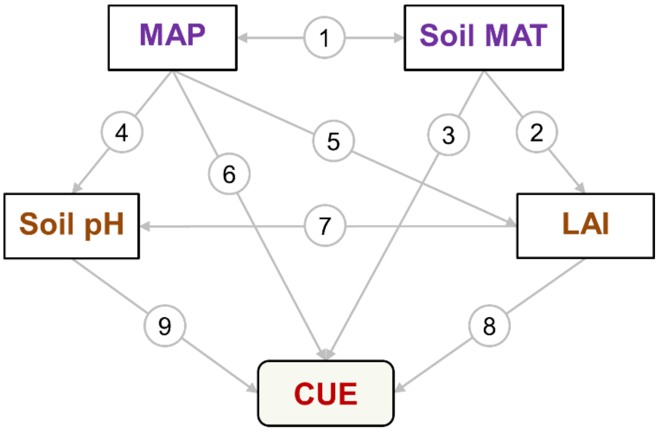

#	Factor	Rationale
1	MAP ⟷ Soil MAT	It is generally assumed that the climate variables interact with each other.
2	Soil MAT ⟶ LAI	Temperature is well known to affect plant productivity in terrestrial ecosystems.
3	Soil MAT ⟶ CUE	Temperature can affect CUE by driving microbial activity and metabolic rate. Hotter terrestrial ecosystems often support higher microbial activity and lower CUE.
4	MAP ⟶ Soil pH	Precipitation can affect the weathering of the parent material and the process of cationic reaction, the amount of litter, and decomposition, which affect soil pH.
5	MAP ⟶ LAI	Precipitation is well known to control plant productivity in terrestrial ecosystems.
6	MAP ⟶ CUE	Precipitation can affect CUE by altering microbial taxa, activity, and metabolic rate. Humid terrestrial ecosystems often support higher microbial activity and lower CUE.
7	LAI ⟶ Soil pH	Plant productivity can affect soil pH through the input of litter and root exudates into the soil.
8	LAI ⟶ CUE	Plant productivity provides the main C source for soil microorganisms, and thus controls CUE. Ecosystems with higher productivity generally have a lower CUE.
9	Soil pH ⟶ CUE	Soil pH can affect CUE by influencing microbial composition and physiological processes.

Fig. S5 The priori pathway modeling including the direct and indirect effects of the four environmental factors we selected on microbial carbon use efficiency (CUE).

Table S4

Detailed results of partial least squares path modeling for the tropical zone.

MODEL SPECIFICATION

1	Number of Cases	590
2	Latent Variables	5
3	Manifest Variables	5
4	Scale of Data	Standardized Data
5	Non-Metric PLS	FALSE
6	Weighting Scheme	centroid
7	Tolerance Crit	1.00E-06
8	Max Num Iters	100
9	Convergence Iters	2
10	Bootstrapping	FALSE
11	Bootstrap samples	NULL

BLOCKS DEFINITION

	Block	Type	Size	Mode
1	MAP	Exogenous	1	A
2	Soil MAT	Exogenous	1	A
3	LAI	Endogenous	1	A
4	Soil pH	Endogenous	1	A
5	CUE_{ST}	Endogenous	1	A

BLOCKS UNIDIMENSIONALITY

	Mode	MVs	C.alpha	DG.rho	eig.1st	eig.2nd
MAP	A	1	1	1	1	0
Soil MAT	A	1	1	1	1	0
LAI	A	1	1	1	1	0
Soil pH	A	1	1	1	1	0
CUE_{ST}	A	1	1	1	1	0

OUTER MODEL

		weight	loading	communality	redundancy
MAP					
1	MAP	1	1	1	0
Soil MAT					
2	Soil MAT	1	1	1	0
LAI					
3	LAI	1	1	1	0.045
Soil pH					
4	Soil pH	1	1	1	0.054
CUE_{ST}					
5	CUE_{ST}	1	1	1	0.096

CROSS LOADINGS

		MAP	Soil MAT	LAI	Soil pH	CUE_{ST}
MAP 1	MAP	1.000	-0.040	0.160	-0.140	-0.031
Soil MAT 2	Soil MAT	-0.040	1.000	0.131	0.182	0.014
LAI 3	LAI	0.160	0.131	1.000	-0.205	-0.081
Soil pH 4	Soil pH	-0.140	0.182	-0.205	1.000	-0.261
CUE_{ST} 5	CUE_{ST}	-0.031	0.014	-0.081	-0.261	1.000

INNER MODEL

$LAI

	Estimate	Std. Error	t value	Pr(>|t|)
Intercept	-3.64E-15	0.0403	-9.02E-14	1.00E+00
MAP	1.66E-01	0.0404	4.11E+00	4.62E-05
Soil MAT	1.37E-01	0.0404	3.40E+00	7.26E-04

$Soil pH

	Estimate	Std. Error	t value	Pr(>|t|)
Intercept	-7.32E-16	0.0402	-1.82E-14	1.00E+00
MAP	-1.10E-01	0.0407	-2.71E+00	6.91E-03
LAI	-1.87E-01	0.0407	-4.60E+00	5.26E-06

$$CUE_{ST}$

	Estimate	Std. Error	t value	Pr(>|t|)
Intercept	-5.25E-16	0.0393	-1.33E-14	1.00E+00
MAP	-4.72E-02	0.0401	-1.18E+00	2.39E-01
Soil MAT	8.82E-02	0.0406	2.17E+00	3.02E-02
LAI	-1.49E-01	0.0412	-3.62E+00	3.22E-04
Soil pH	-3.14E-01	0.0413	-7.59E+00	1.26E-13

CORRELATIONS BETWEEN LVs

	MAP	Soil MAT	LAI	Soil pH	CUE_{ST}
MAP	1.000	-0.040	0.160	-0.140	-0.031
Soil MAT	-0.040	1.000	0.131	0.182	0.014
LAI	0.160	0.131	1.000	-0.205	-0.081
Soil pH	-0.140	0.182	-0.205	1.000	-0.261
CUE_{ST}	-0.031	0.014	-0.081	-0.261	1.000

SUMMARY INNER MODEL

	Type	R2	Block_Communality	Mean_Redundancy	AVE
MAP	Exogenous	0	1	0	1

Soil MAT	Exogenous	0	1	0	1
LAI	Endogenous	0.045	1	0.045	1
Soil pH	Endogenous	0.054	1	0.054	1
CUE_{ST}	Endogenous	0.096	1	0.096	1

GOODNESS-OF-FIT

[1]	NaN

TOTAL EFFECTS

	relationships	direct	indirect	total
1	MAP -> Soil MAT	0.000	0.000	0.000
2	MAP-> LAI	0.166	0.000	0.166
3	MAP -> Soil pH	-0.110	-0.031	-0.141
4	MAP -> CUE_{ST}	-0.047	0.020	-0.028
5	Soil MAT -> LAI	0.137	0.000	0.137
6	Soil MAT -> Soil pH	0.000	-0.026	-0.026
7	Soil MAT -> CUE_{ST}	0.088	-0.012	0.076
8	LAI -> Soil pH	-0.187	0.000	-0.187
9	LAI -> CUE_{ST}	-0.149	0.059	-0.090
10	Soil pH -> CUE_{ST}	-0.314	0.000	-0.314

Note: MAP, mean annual precipitation; Soil MAT, mean annual soil temperature; LAI, leaf area index. Since each latent variable in our models contains only one manifest variable, the modeling run does not generate values of the goodness of fit.

Table S5

Detailed results of partial least squares path modeling for the arid zone.

MODEL SPECIFICATION

1	Number of Cases	129
2	Latent Variables	5
3	Manifest Variables	5
4	Scale of Data	Standardized Data
5	Non-Metric PLS	FALSE
6	Weighting Scheme	centroid
7	Tolerance Crit	1.00E-06
8	Max Num Iters	100
9	Convergence Iters	2
10	Bootstrapping	FALSE
11	Bootstrap samples	NULL

BLOCKS DEFINITION

	Block	Type	Size	Mode
1	MAP	Exogenous	1	A
2	Soil MAT	Exogenous	1	A
3	LAI	Endogenous	1	A
4	Soil pH	Endogenous	1	A
5	CUE_{ST}	Endogenous	1	A

BLOCKS UNIDIMENSIONALITY

	Mode	MVs	C.alpha	DG.rho	eig.1st	eig.2nd
MAP	A	1	1	1	1	0
Soil MAT	A	1	1	1	1	0
LAI	A	1	1	1	1	0
Soil pH	A	1	1	1	1	0
CUE_{ST}	A	1	1	1	1	0

OUTER MODEL

		weight	loading	communality	redundancy
MAP					
1	MAP	1	1	1	0
Soil MAT					
2	Soil MAT	1	1	1	0
LAI					
3	LAI	1	1	1	0.273
Soil pH					
4	Soil pH	1	1	1	0.226
CUE_{ST}					
5	CUE_{ST}	1	1	1	0.567

CROSSLOADINGS

		MAP	Soil MAT	LAI	Soil pH	CUE_{ST}
MAP 1	MAP	1.000	0.654	0.516	-0.473	-0.721
Soil MAT 2	Soil MAT	0.654	1.000	0.398	-0.296	-0.488
LAI 3	LAI	0.516	0.398	1.000	-0.286	-0.486
Soil pH 4	Soil pH	-0.473	-0.296	-0.286	1.000	0.496
CUE_{ST} 5	CUE_{ST}	-0.721	-0.488	-0.486	0.496	1.000

INNER MODEL

$LAI				
	Estimate	Std. Error	t value	Pr(>|t|)
Intercept	-1.36E-16	0.076	-1.79E-15	1.00E+00
MAP	4.48E-01	0.1	4.46E+00	1.79E-05
Soil MAT	1.05E-01	0.1	1.04E+00	2.99E-01
**$Soil pH**				
	Estimate	Std. Error	t value	Pr(>|t|)
Intercept	5.92E-17	0.0784	7.55E-16	1.00E+00
MAP	-4.44E-01	0.0915	-4.85E+00	3.64E-06
LAI	-5.66E-02	0.0915	-6.18E-01	5.38E-01
**$$CUE_{ST}$**				
	Estimate	Std. Error	t value	Pr(>|t|)
Intercept	1.64E-16	0.0591	2.77E-15	1.00E+00
MAP	-5.45E-01	0.0896	-6.08E+00	1.37E-08
Soil MAT	-1.78E-02	0.0785	-2.27E-01	8.21E-01
LAI	-1.42E-01	0.0695	-2.04E+00	4.30E-02
Soil pH	1.93E-01	0.0672	2.87E+00	4.84E-03

CORRELATIONS BETWEEN LVs

	MAP	Soil MAT	LAI	Soil pH	CUE_{ST}
MAP	1.000	0.654	0.516	-0.473	-0.721
Soil MAT	0.654	1.000	0.398	-0.296	-0.488
LAI	0.516	0.398	1.000	-0.286	-0.486
Soil pH	-0.473	-0.296	-0.286	1.000	0.496
CUE_{ST}	-0.721	-0.488	-0.486	0.496	1.000

SUMMARY INNER MODEL

	Type	R2	Block_Communality	Mean_Redundancy	AVE
MAP	Exogenous	0	1	0	1

Soil MAT	Exogenous	0	1	0	1
LAI	Endogenous	0.273	1	0.273	1
Soil pH	Endogenous	0.226	1	0.226	1
CUE_{ST}	Endogenous	0.567	1	0.567	1

GOODNESS-OF-FIT

[1]	NaN

TOTAL EFFECTS

	relationships	direct	indirect	total
1	MAP -> Soil MAT	0.000	0.000	0.000
2	MAP-> LAI	0.448	0.000	0.448
3	MAP -> Soil pH	-0.444	-0.025	-0.469
4	MAP -> CUE_{ST}	-0.545	-0.154	-0.699
5	Soil MAT -> LAI	0.105	0.000	0.105
6	Soil MAT -> Soil pH	0.000	-0.006	-0.006
7	Soil MAT -> CUE_{ST}	-0.018	-0.016	-0.034
8	LAI -> Soil pH	-0.057	0.000	-0.057
9	LAI -> CUE_{ST}	-0.142	-0.011	-0.153
10	Soil pH -> CUE_{ST}	0.193	0.000	0.193

Note: MAP, mean annual precipitation; Soil MAT, mean annual soil temperature; LAI, leaf area index. Since each latent variable in our models contains only one manifest variable, the modeling run does not generate values of the goodness of fit.

Table S6

Detailed results of partial least squares path modeling for the temperate zone.

MODEL SPECIFICATION

1	Number of Cases	114
2	Latent Variables	5
3	Manifest Variables	5
4	Scale of Data	Standardized Data
5	Non-Metric PLS	FALSE
6	Weighting Scheme	centroid
7	Tolerance Crit	1.00E-06
8	Max Num Iters	100
9	Convergence Iters	2
10	Bootstrapping	FALSE
11	Bootstrap samples	NULL

BLOCKS DEFINITION

	Block	Type	Size	Mode
1	MAP	Exogenous	1	A
2	Soil MAT	Exogenous	1	A
3	LAI	Endogenous	1	A
4	Soil pH	Endogenous	1	A
5	CUE_{ST}	Endogenous	1	A

BLOCKS UNIDIMENSIONALITY

	Mode	MVs	C.alpha	DG.rho	eig.1st	eig.2nd
MAP	A	1	1	1	1	0
Soil MAT	A	1	1	1	1	0
LAI	A	1	1	1	1	0
Soil pH	A	1	1	1	1	0
CUE_{ST}	A	1	1	1	1	0

OUTER MODEL

		weight	loading	communality	redundancy
MAP					
1	MAP	1	1	1	0
Soil MAT					
2	Soil MAT	1	1	1	0
LAI					
3	LAI	1	1	1	0.120
Soil pH					
4	Soil pH	1	1	1	0.043
CUE_{ST}					
5	CUE_{ST}	1	1	1	0.265

CROSSLOADINGS

		MAP	Soil MAT	LAI	Soil pH	CUE_{ST}
MAP 1	MAP	1.000	-0.103	0.329	-0.149	-0.072
Soil MAT 2	Soil MAT	-0.103	1.000	0.073	0.261	0.271
LAI 3	LAI	0.329	0.073	1.000	-0.183	-0.174
Soil pH 4	Soil pH	-0.149	0.261	-0.183	1.000	0.478
CUE_{ST} 5	CUE_{ST}	-0.072	0.271	-0.174	0.478	1.000

INNER MODEL

$LAI				
	Estimate	Std. Error	t value	Pr(>|t|)
Intercept	7.58E-17	0.0891	8.51E-16	1.00E+00
MAP	3.40E-01	0.0895	3.80E+00	2.41E-04
Soil MAT	1.08E-01	0.0895	1.20E+00	2.32E-01
$Soil pH				
	Estimate	Std. Error	t value	Pr(>|t|)
Intercept	-1.66E-17	0.0929	-1.79E-16	1.00E+00
MAP	-9.99E-02	0.0983	-1.02E+00	3.12E-01
LAI	-1.51E-01	0.0983	-1.53E+00	1.29E-01
$$CUE_{ST}$				
	Estimate	Std. Error	t value	Pr(>|t|)
Intercept	7.34E-17	0.0821	8.94E-16	1.00E+00
MAP	4.97E-02	0.0879	5.66E-01	5.73E-01
Soil MAT	1.77E-01	0.0863	2.05E+00	4.25E-02
LAI	-1.27E-01	0.089	-1.43E+00	1.56E-01
Soil pH	4.15E-01	0.0872	4.77E+00	5.85E-06

CORRELATIONS BETWEEN LVs

	MAP	Soil MAT	LAI	Soil pH	CUE_{ST}
MAP	1.000	-0.103	0.329	-0.149	-0.072
Soil MAT	-0.103	1.000	0.073	0.261	0.271
LAI	0.329	0.073	1.000	-0.183	-0.174
Soil pH	-0.149	0.261	-0.183	1.000	0.478
CUE_{ST}	-0.072	0.271	-0.174	0.477	1.000

SUMMARY INNER MODEL

	Type	R2	Block_Communality	Mean_Redundancy	AVE
MAP	Exogenous	0	1	0	1

Soil MAT	Exogenous	0	1	0	1
LAI	Endogenous	0.120	1	0.120	1
Soil pH	Endogenous	0.043	1	0.043	1
CUE_{ST}	Endogenous	0.265	1	0.265	1

GOODNESS-OF-FIT

[1]	NaN

TOTAL EFFECTS

	relationships	direct	indirect	total
1	MAP -> Soil MAT	0.000	0.000	0.000
2	MAP-> LAI	0.340	0.000	0.340
3	MAP -> Soil pH	-0.100	-0.051	-0.151
4	MAP -> CUE_{ST}	0.050	-0.106	-0.056
5	Soil MAT -> LAI	0.108	0.000	0.108
6	Soil MAT -> Soil pH	0.000	-0.016	-0.016
7	Soil MAT -> CUE_{ST}	0.177	-0.020	0.157
8	LAI -> Soil pH	-0.151	0.000	-0.151
9	LAI -> CUE_{ST}	-0.127	-0.063	-0.190
10	Soil pH -> CUE_{ST}	0.415	0.000	0.415

Note: MAP, mean annual precipitation; Soil MAT, mean annual soil temperature; LAI, leaf area index. Since each latent variable in our models contains only one manifest variable, the modeling run does not generate values of the goodness of fit.

Table S7

Detailed results of partial least squares path modeling for the cold zone.

MODEL SPECIFICATION

1	Number of Cases	261
2	Latent Variables	5
3	Manifest Variables	5
4	Scale of Data	Standardized Data
5	Non-Metric PLS	FALSE
6	Weighting Scheme	centroid
7	Tolerance Crit	1.00E-06
8	Max Num Iters	100
9	Convergence Iters	2
10	Bootstrapping	FALSE
11	Bootstrap samples	NULL

BLOCKS DEFINITION

	Block	Type	Size	Mode
1	MAP	Exogenous	1	A
2	Soil MAT	Exogenous	1	A
3	LAI	Endogenous	1	A
4	Soil pH	Endogenous	1	A
5	CUE_{ST}	Endogenous	1	A

BLOCKS UNIDIMENSIONALITY

	Mode	MVs	C.alpha	DG.rho	eig.1st	eig.2nd
MAP	A	1	1	1	1	0
Soil MAT	A	1	1	1	1	0
LAI	A	1	1	1	1	0
Soil pH	A	1	1	1	1	0
CUE_{ST}	A	1	1	1	1	0

OUTER MODEL

		weight	loading	communality	redundancy
MAP					
1	MAP	1	1	1	0
Soil MAT					
2	Soil MAT	1	1	1	0
LAI					
3	LAI	1	1	1	0.315
Soil pH					
4	Soil pH	1	1	1	0.288
CUE_{ST}					
5	CUE_{ST}	1	1	1	0.238

CROSSLOADINGS

		MAP	Soil MAT	LAI	Soil pH	CUE$_{ST}$
MAP 1	MAP	1.000	-0.023	0.554	-0.477	-0.370
Soil MAT 2	Soil MAT	-0.023	1.000	-0.100	0.342	0.133
LAI 3	LAI	0.554	-0.100	1.000	-0.470	-0.458
Soil pH 4	Soil pH	-0.477	0.342	-0.470	1.000	0.281
CUE$_{ST}$ 5	CUE$_{ST}$	-0.370	0.133	-0.458	0.281	1.000

INNER MODEL

$LAI

	Estimate	Std. Error	t value	Pr(>|t|)
Intercept	2.68E-16	0.0515	5.21E-15	1.00E+00
MAP	5.52E-01	0.0515	1.07E+01	2.09E-22
Soil MAT	-8.75E-02	0.0515	-1.70E+00	9.08E-02

$Soil pH

	Estimate	Std. Error	t value	Pr(>|t|)
Intercept	3.82E-16	0.0525	7.28E-15	1.00E+00
MAP	-3.13E-01	0.0631	-4.95E+00	1.33E-06
LAI	-2.96E-01	0.0631	-4.70E+00	4.29E-06

CUE_{ST}$

	Estimate	Std. Error	t value	Pr(>|t|)
Intercept	7.74E-17	0.0546	1.42E-15	1.00E+00
MAP	-1.71E-01	0.0694	-2.46E+00	1.45E-02
Soil MAT	9.26E-02	0.0589	1.57E+00	1.17E-01
LAI	-3.53E-01	0.0683	-5.16E+00	4.87E-07
Soil pH	2.16E-03	0.0694	3.11E-02	9.75E-01

CORRELATIONS BETWEEN LVs

	MAP	Soil MAT	LAI	Soil pH	CUE$_{ST}$
MAP	1.000	-0.023	0.554	-0.477	-0.370
Soil MAT	-0.023	1.000	-0.100	0.342	0.133
LAI	0.554	-0.100	1.000	-0.470	-0.458
Soil pH	-0.477	0.342	-0.470	1.000	0.281
CUE$_{ST}$	-0.370	0.133	-0.458	0.281	1.000

SUMMARY INNER MODEL

	Type	R2	Block_Communality	Mean_Redundancy	AVE
MAP	Exogenous	0	1	0	1

Soil MAT	Exogenous	0	1	0	1
LAI	Endogenous	0.315	1	0.315	1
Soil pH	Endogenous	0.288	1	0.288	1
CUE_{ST}	Endogenous	0.238	1	0.238	1

GOODNESS-OF-FIT

[1]	NaN

TOTAL EFFECTS

	relationships	direct	indirect	total
1	MAP -> Soil MAT	0.000	0.000	0.000
2	MAP-> LAI	0.552	0.000	0.552
3	MAP -> Soil pH	-0.313	-0.164	-0.476
4	MAP -> CUE_{ST}	-0.171	-0.196	-0.367
5	Soil MAT -> LAI	-0.087	0.000	-0.087
6	Soil MAT -> Soil pH	0.000	0.026	0.026
7	Soil MAT -> CUE_{ST}	0.093	0.031	0.123
8	LAI -> Soil pH	-0.296	0.000	-0.296
9	LAI -> CUE_{ST}	-0.353	-0.001	-0.353
10	Soil pH -> CUE_{ST}	0.002	0.000	0.002

Note: MAP, mean annual precipitation; Soil MAT, mean annual soil temperature; LAI, leaf area index. Since each latent variable in our models contains only one manifest variable, the modeling run does not generate values of the goodness of fit.

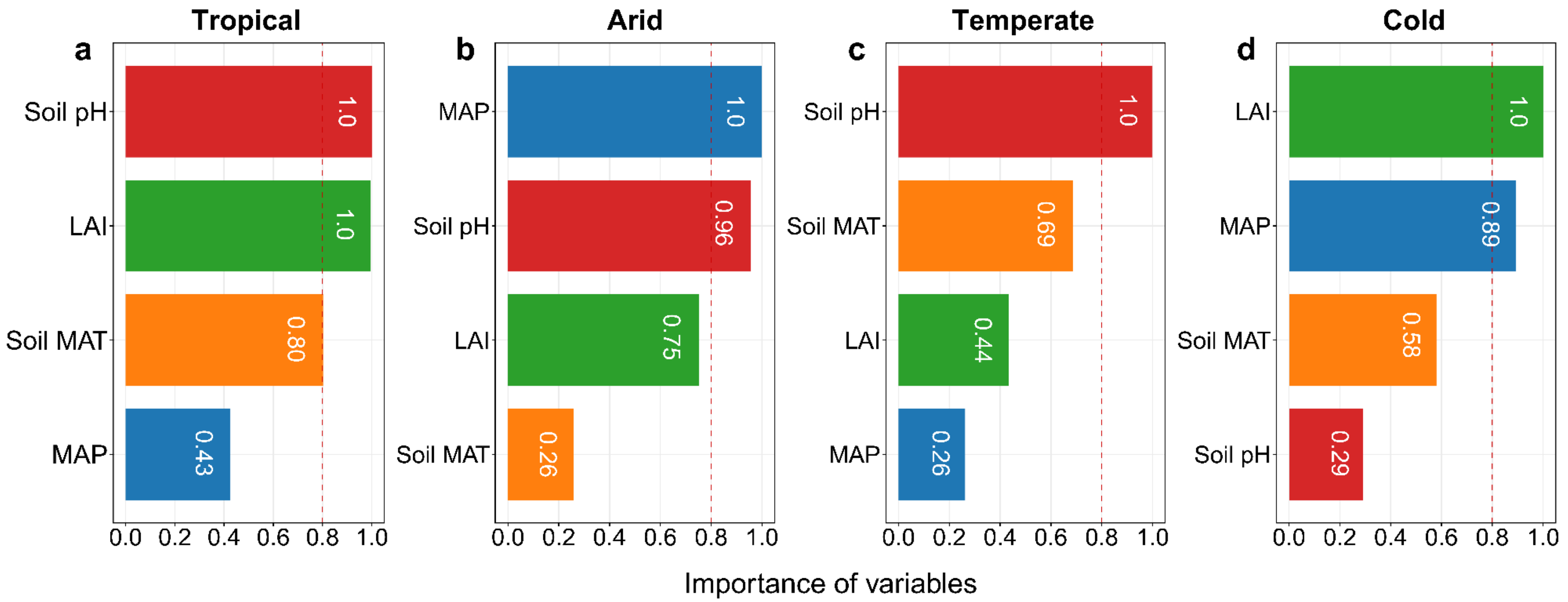

Fig. S6 Effects of four selected environmental variables on microbial carbon use efficiency (CUE_{ST}) using the analysis of mixed-effects model selection across climate zones. The values of the bars are the relative importance of the variables regulating the variations in CUE_{ST}, which were estimated using the sum of Akaike weights based on the model selection analysis using the corrected Akaike's information criteria. A threshold value of 0.8 was set to identify the most important variables. MAP, mean annual precipitation; Soil MAT, mean annual soil temperature; LAI, leaf area index.

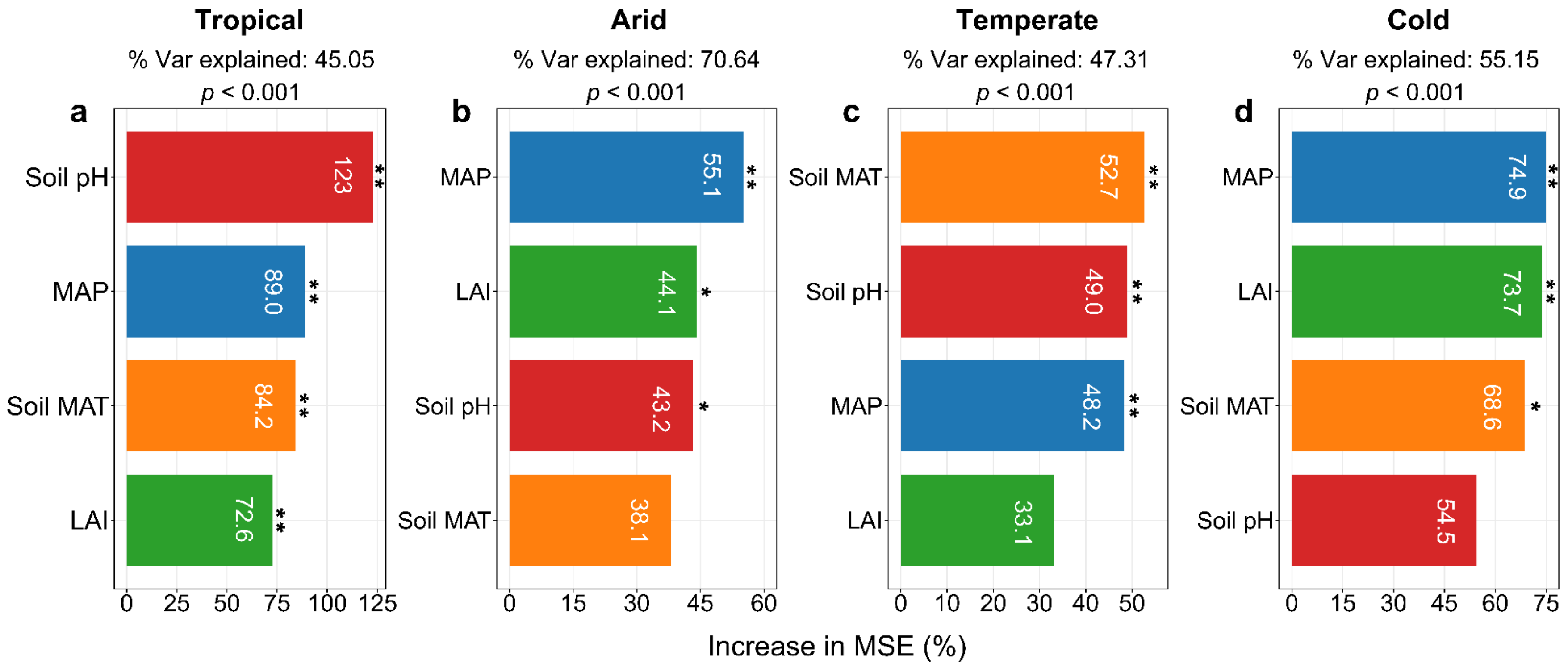

Fig. S7 Effects of four selected environmental variables on microbial carbon use efficiency (CUE_{ST}) using random-forest models across climate zones. MAP, mean annual precipitation; Soil MAT, mean annual soil temperature; LAI, leaf area index. *, $p < 0.05$; **, $p < 0.01$.

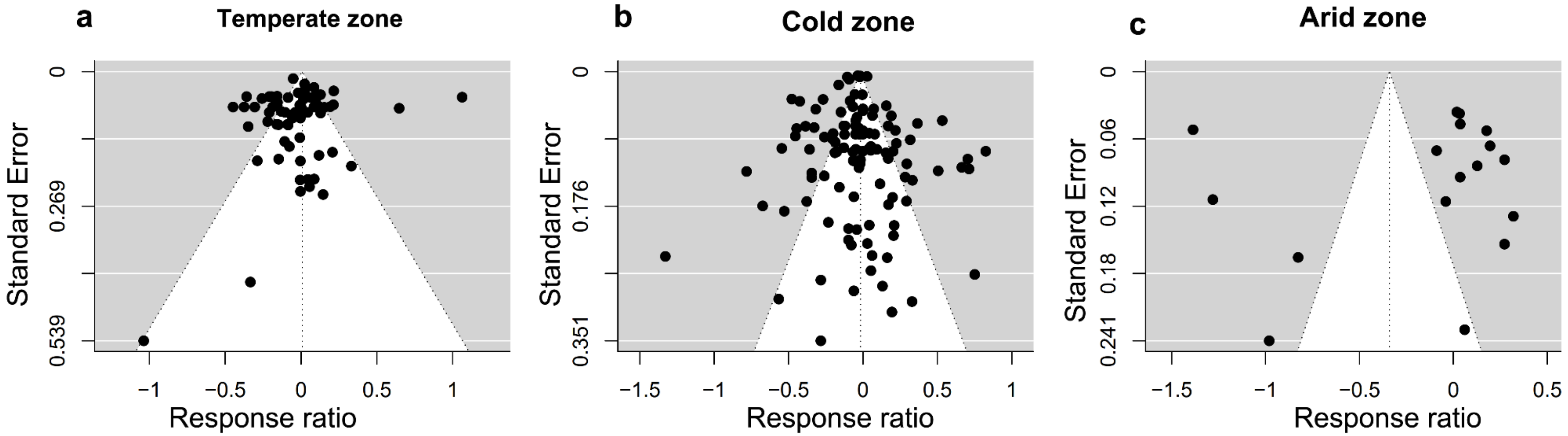

Fig. S8 Funnel plot testing the publication bias of the natural logarithmic response ratio of microbial carbon use efficiency (CUE-isotope) measured with ^{13}C- or ^{18}O-labeled approaches to six manipulative factors around the world.

Reference list of the dataset of mate-analysis

Note: *The list includes 41 studies that measure microbial carbon use efficiency (CUE-isotope) using ^{13}C- or ^{18}O-labeled approaches.*

Canarini, A., Schmidt, H., Fuchslueger, L., Martin, V., Herbold, C. W., Zezula, D., ... & Richter, A. (2021). Ecological memory of recurrent drought modifies soil processes via changes in soil microbial community. *Nature Communications*, *12*(1), 1-14.

Chen, Y., Zhang, Y., Bai, E., Piao, S., Chen, N., Zhao, G., ... & Zhu, Y. (2022). The stimulatory effect of elevated CO_2 on soil respiration is unaffected by N addition. *Science of the Total Environment*, *813*, 151907.

Cheng, H., Hill, P. W., Bastami, M. S., & Jones, D. L. (2017). Biochar stimulates the decomposition of simple organic matter and suppresses the decomposition of complex organic matter in a sandy loam soil. *GCB Bioenergy*, *9*(6), 1110-1121.

Cui, Y., Moorhead, D. L., Wang, X., Xu, M., Wang, X., Wei, X., ... & Fang, L. (2022). Decreasing microbial phosphorus limitation increases soil carbon release. *Geoderma*, *419*, 115868.

Dijkstra, P., Thomas, S. C., Heinrich, P. L., Koch, G. W., Schwartz, E., & Hungate, B. A. (2011). Effect of temperature on metabolic activity of intact microbial communities: evidence for altered metabolic pathway activity but not for increased maintenance respiration and reduced carbon use efficiency. *Soil Biology and Biochemistry*, *43*(10), 2023-2031.

Dove, N. C., Torn, M. S., Hart, S. C., & Taş, N. (2021). Metabolic capabilities mute positive response to direct and indirect impacts of warming throughout the soil profile. *Nature Communications*, *12*(1), 1-13.

Fang, Y., Singh, B. P., Collins, D., Li, B., Zhu, J., & Tavakkoli, E. (2018). Nutrient supply enhanced wheat residue-carbon mineralization, microbial growth, and microbial carbon-use efficiency when residues were supplied at high rate in contrasting soils. *Soil Biology and Biochemistry*, *126*, 168-178.

Feng, X., Qin, S., Zhang, D., Chen, P., Hu, J., Wang, G., ... & Chen, L. (2022). Nitrogen input enhances microbial carbon use efficiency by altering plant–microbe–mineral interactions. *Global Change Biology*, *28*(16), 4845-4860.

Fisk, L. M., Barton, L., Jones, D. L., Glanville, H. C., & Murphy, D. V. (2015). Root exudate carbon mitigates nitrogen loss in a semi-arid soil. *Soil Biology and Biochemistry*, *88*, 380-389.

Frey, S. D., Lee, J., Melillo, J. M., & Six, J. (2013). The temperature response of soil microbial efficiency and its feedback to climate. *Nature Climate Change*, *3*(4), 395-398.

Fuchslueger, L., Wild, B., Mooshammer, M., Takriti, M., Kienzl, S., Knoltsch, A., ... & Richter, A. (2019). Microbial carbon and nitrogen cycling responses to drought and temperature in differently managed mountain grasslands. *Soil Biology and Biochemistry*, *135*, 144-153.

Geyer, K. M., Dijkstra, P., Sinsabaugh, R., & Frey, S. D. (2019). Clarifying the interpretation of carbon use efficiency in soil through methods comparison. *Soil Biology and Biochemistry*, *128*, 79-88.

Hicks, L. C., Leizeaga, A., Rousk, K., Michelsen, A., & Rousk, J. (2020). Simulated rhizosphere deposits induce microbial N-mining that may accelerate shrubification in the subarctic. *Ecology*, *101*(9), e03094.

Hicks, L. C., Yuan, M., Brangarí, A., Rousk, K., & Rousk, J. (2022). Increased Above-and Belowground Plant Input Can Both Trigger Microbial Nitrogen Mining in Subarctic Tundra Soils. *Ecosystems*, *25*(1), 105-121.

Li, J., Sang, C., Yang, J., Qu, L., Xia, Z., Sun, H., ... & Wang, C. (2021). Stoichiometric imbalance and microbial community regulate microbial elements use efficiencies under nitrogen addition. *Soil Biology and Biochemistry*, *156*, 108207.

Li, J. H., Zhang, R., Cheng, B. H., Ye, L. F., Li, W. J., & Shi, X. M. (2021). Effects of nitrogen and phosphorus additions on decomposition and accumulation of soil organic carbon in alpine meadows on the Tibetan Plateau. *Land Degradation & Development*, *32*(3), 1467-1477.

Liu, W., Qiao, C., Yang, S., Bai, W., & Liu, L. (2018). Microbial carbon use efficiency and priming effect regulate soil carbon storage under nitrogen deposition by slowing soil organic matter decomposition. *Geoderma*, *332*, 37-44.

Liu, X. J. A., Pold, G., Domeignoz-Horta, L. A., Geyer, K. M., Caris, H., Nicolson, H., ... & DeAngelis, K. M. (2021). Soil aggregate-mediated microbial responses to long-term warming. *Soil Biology and Biochemistry*, *152*, 108055.

Liu, Z., Wu, X., Liu, W., Bian, R., Ge, T., Zhang, W., ... & Pan, G. (2020). Greater microbial carbon use efficiency and carbon sequestration in soils: Amendment of biochar versus crop straws. *GCB Bioenergy*, *12*(12), 1092-1103.

Luo, R., Kuzyakov, Y., Liu, D., Fan, J., Luo, J., Lindsey, S., ... & Ding, W. (2020). Nutrient addition reduces carbon sequestration in a Tibetan grassland soil: Disentangling microbial and physical controls. *Soil Biology and Biochemistry*, *144*, 107764.

Ma, Q., Wen, Y., Pan, W., Macdonald, A., Hill, P. W., Chadwick, D. R., ... & Jones, D. L. (2020). Soil carbon, nitrogen, and sulphur status affects the metabolism of organic S but not its uptake by microorganisms. *Soil Biology and Biochemistry*, *149*, 107943.

Mehnaz, K. R., Corneo, P. E., Keitel, C., & Dijkstra, F. A. (2019). Carbon and phosphorus addition effects on microbial carbon use efficiency, soil organic matter priming, gross nitrogen mineralization and nitrous oxide emission from soil. *Soil Biology and Biochemistry*, *134*, 175-186.

Miao, Y., Niu, Y., Luo, R., Li, Y., Zheng, H., Kuzyakov, Y., ... & Ding, W. (2021). Lower microbial carbon use efficiency reduces cellulose-derived carbon retention in soils amended with compost versus mineral fertilizers. *Soil Biology and Biochemistry*, *156*, 108227.

Morris, K. A., Richter, A., Migliavacca, M., & Schrumpf, M. (2022). Growth of soil microbes is not limited by the availability of nitrogen and phosphorus in a Mediterranean oak-savanna. *Soil Biology and Biochemistry*, *169*, 108680.

Poeplau, C., Helfrich, M., Dechow, R., Szoboszlay, M., Tebbe, C. C., Don, A., ... & Geerts, R. (2019). Increased microbial anabolism contributes to soil carbon sequestration by mineral fertilization in temperate grasslands. *Soil Biology and Biochemistry*, *130*, 167-176.

Riggs, C. E., & Hobbie, S. E. (2016). Mechanisms driving the soil organic matter decomposition response to nitrogen enrichment in grassland soils. *Soil Biology and Biochemistry*, *99*, 54-65.

Schindlbacher, A., Schnecker, J., Takriti, M., Borken, W., & Wanek, W. (2015). Microbial physiology and soil CO_2 efflux after 9 years of soil warming in a temperate forest–no indications for thermal adaptations. *Global Change Biology*, *21*(11), 4265-4277.

Silva-Sánchez, A., Soares, M., & Rousk, J. (2019). Testing the dependence of microbial growth and carbon use efficiency on nitrogen availability, pH, and organic matter quality. *Soil Biology and Biochemistry*, *134*, 25-35.

Simon, E., Canarini, A., Martin, V., Séneca, J., Böckle, T., Reinthaler, D., ... & Richter, A. (2020). Microbial growth and carbon use efficiency show seasonal responses in a multifactorial climate change experiment. *Communications Biology*, *3*(1), 1-10.

Spohn, M., Pötsch, E. M., Eichorst, S. A., Woebken, D., Wanek, W., & Richter, A. (2016). Soil microbial carbon use efficiency and biomass turnover in a long-term fertilization experiment in a temperate grassland. *Soil Biology and Biochemistry, 97,* 168-175.

Tang, H., Li, C., Wen, L., Li, W., Shi, L., Cheng, K., & Xiao, X. (2020). Microbial carbon source utilization in rice rhizosphere and non-rhizosphere soils in a 34-year fertilized paddy field. *Journal of Basic Microbiology*, *60*(11-12), 1004-1013.

Ullah, M. R., Carrillo, Y., & Dijkstra, F. A. (2021). Drought-induced and seasonal variation in carbon use efficiency is associated with fungi: bacteria ratio and enzyme production in a grassland ecosystem. *Soil Biology and Biochemistry*, *155*, 108159.

Walker, T. W., Kaiser, C., Strasser, F., Herbold, C. W., Leblans, N. I., Woebken, D., ... & Richter, A. (2018). Microbial temperature sensitivity and biomass change explain soil carbon loss with warming. *Nature Climate Change*, *8*(10), 885-889.

Wang, M., Dungait, J. A., Wei, X., Ge, T., Hou, R., Ouyang, Z., ... & Tian, J. (2022). Long-term warming increased microbial carbon use efficiency and turnover rate under conservation tillage system. *Soil Biology and Biochemistry*, *172*, 108770.

Wang, W., Zhu, W., Li, X., & Ma, S. (2022). Long-term nitrogen addition increased soil microbial carbon use efficiency in subalpine forests on the eastern edge of the Qinghai-Tibet Plateau. *Plant and Soil*.

Widdig, M., Schleuss, P. M., Biederman, L. A., Borer, E. T., Crawley, M. J., Kirkman, K. P., ... & Spohn, M. (2020). Microbial carbon use efficiency in grassland soils subjected to nitrogen and phosphorus additions. *Soil Biology and Biochemistry*, *146*, 107815.

Xiao, Q., Huang, Y., Wu, L., Tian, Y., Wang, Q., Wang, B., ... & Zhang, W. (2021). Long-term manuring increases microbial carbon use efficiency and mitigates priming effect via alleviated soil acidification and resource limitation. *Biology and Fertility of Soils*, *57*(7), 925-934.

Yin, L., Corneo, P. E., Richter, A., Wang, P., Cheng, W., & Dijkstra, F. A. (2019). Variation in rhizosphere priming and microbial growth and carbon use efficiency caused by wheat genotypes and temperatures. *Soil Biology and Biochemistry*, *134,* 54-61.

Zhran, M., Ge, T., Tong, Y., Deng, Y., Wei, X., Lynn, T. M., ... & Gunina, A. (2021). Assessment of depth-dependent microbial carbon-use efficiency in long-term fertilized paddy soil using an $^{18}O\text{-}H_2O$ approach. *Land Degradation & Development*, *32*(1), 199-207.

Zhu, E., Cao, Z., Jia, J., Liu, C., Zhang, Z., Wang, H., ... & Feng, X. (2021). Inactive and inefficient: Warming and drought effect on microbial carbon processing in alpine grassland at depth. *Global Change Biology*, *27*(10), 2241-2253.

Ziegler, S. E., & Billings, S. A. (2011). Soil nitrogen status as a regulator of carbon substrate flows through microbial communities with elevated CO_2. *Journal of Geophysical Research: Biogeosciences*, *116*(G1).
